# Telomerase governs immunomodulatory properties of mesenchymal stem cells by regulating FAS ligand expression

**DOI:** 10.1002/emmm.201303000

**Published:** 2014-01-13

**Authors:** Chider Chen, Kentaro Akiyama, Takayoshi Yamaza, Yong-Ouk You, Xingtian Xu, Bei Li, Yimin Zhao, Songtao Shi

**Affiliations:** 1 Center for Craniofacial Molecular Biology, University of Southern CaliforniaLos Angeles, CA, USA; 2 Department of Oral Rehabilitation and Regenerative Medicine, Okayama University Graduate School of Medicine, Dentistry, and Pharmaceutical ScienceKita-ku, Okayama, Japan; 3 Department of Molecular Cell Biology and Oral Anatomy, Kyushu University Graduate School of Dental ScienceFukuoka, Japan; 4 School of Stomatology, Fourth Military Medical UniversityXi'an, Shanxi, China

**Keywords:** immunomodulation, mesenchymal stem cell, telomerase

## Abstract

Bone marrow mesenchymal stem cells (BMMSCs) are capable of differentiating into multiple cell types and regulating immune cell response. However, the mechanisms that govern the immunomodulatory properties of BMMSCs are still not fully elucidated. Here we show that telomerase-deficient BMMSCs lose their capacity to inhibit T cells and ameliorate the disease phenotype in systemic sclerosis mice. Restoration of telomerase activity by telomerase reverse transcriptase (TERT) transfection in *TERT*^−/−^ BMMSCs rescues their immunomodulatory functions. Mechanistically, we reveal that TERT, combined with β-catenin and BRG1, serves as a transcriptional complex, which binds the FAS ligand (FASL) promoter to upregulate FASL expression, leading to an elevated immunomodulatory function. To test the translational value of these findings in the context of potential clinical therapy, we used aspirin treatment to upregulate telomerase activity in BMMSCs, and found a significant improvement in the immunomodulatory capacity of BMMSCs. Taken together, these findings identify a previously unrecognized role of TERT in improving the immunomodulatory capacity of BMMSCs, suggesting that aspirin treatment is a practical approach to significantly reduce cell dosage in BMMSC-based immunotherapies.

**Subject Categories** Stem Cells; Immunology

## Introduction

Bone marrow mesenchymal stem cells (BMMSCs) are hierarchical postnatal stem cells capable of undergoing self-renewal and multipotent differentiation into osteoblasts, chondrocytes, myelosupportive stroma and adipocytes (Friedenstein *et al*, 1974; Prockop, 1997). BMMSCs are considered to be progenitors of osteoblasts with the capacity to regenerate bone and marrow components *in vivo*. These findings have led to extensive studies using BMMSCs for orthopaedic tissue engineering applications (Kwan *et al*, 2008; Panetta *et al*, 2009). Recently, a growing body of evidence has indicated that BMMSCs produce a variety of cytokines that display profound immunomodulatory properties by inhibiting the proliferation and function of several major types of immune cells, such as natural killer cells, dendritic cells, and both T and B lymphocytes (Aggarwal ' Pittenger, 2005; Nauta ' Fibbe, 2007; Uccelli *et al*, 2007, 2008). These unique properties make BMMSCs a plausible resource for the clinical treatment of immune disorders. To date, systemic infusion of BMMSCs has been successfully used for treating a variety of human diseases, including acute graft-versus-host-disease (GvHD), as well as ameliorating hematopoietic stem cell (HSC) engraftment, systemic lupus erythematosus (SLE), diabetes, rheumatoid arthritis, autoimmune encephalomyelitis, periodontitis, inflammatory bowel disease, sepsis, and systemic sclerosis (Le Blanc *et al*, 2004; Chen *et al*, 2006; Liang *et al*, 2009, 2012; Sun *et al*, 2009; Scuderi *et al*, 2013; Liu *et al*, 2013). A variety of factors, including transforming growth factor β (TGFβ), interleukin-10 (IL-10), prostaglandin E2 (PGE2), nitric oxide (NO), indoleamine 2,3-dioxygenase (IDO), and FAS/FAS ligand (FASL), have been identified as potential regulators of BMMSC-based immunomodulation (Meisel *et al*, 2004; Aggarwal ' Pittenger, 2005; Batten *et al*, 2006; Sato *et al*, 2007; Ren *et al*, 2008; Zhang *et al*, 2008; Park *et al*, 2011; Akiyama *et al*, 2012). However, the precise mechanisms underlying the immunomodulatory properties of BMMSCs remain to be elucidated.

Telomerase reverse transcriptase (TERT) is a nucleoprotein that functions to preserve chromosomal integrity and quell p53-dependent DNA damage, as well as perform DNA repair activity at telomere ends. In the absence of telomerase, continued cell division results in telomere shortening and p53 activation (Maser ' DePinho, 2002; Smogorzewska ' de Lange, 2004). It has been reported that telomerase plays important roles in stem cell self-renewal and stem cell-based tissue regeneration (Yamaza *et al*, 2008; Liu *et al*, 2011), and is highly expressed in prospectively isolated BMMSCs from aspirates of human bone marrow (Gronthos *et al*, 2003). However, the role of TERT in regulating BMMSC-mediated immunomodulation has never been examined considering that TERT is rapidly down regulated in human BMMSCs during *ex vivo* expansion (Shi *et al*, 2002).

## Results

### TERT is associated with BMMSC-mediated immunomodulation

**Figure 1:**
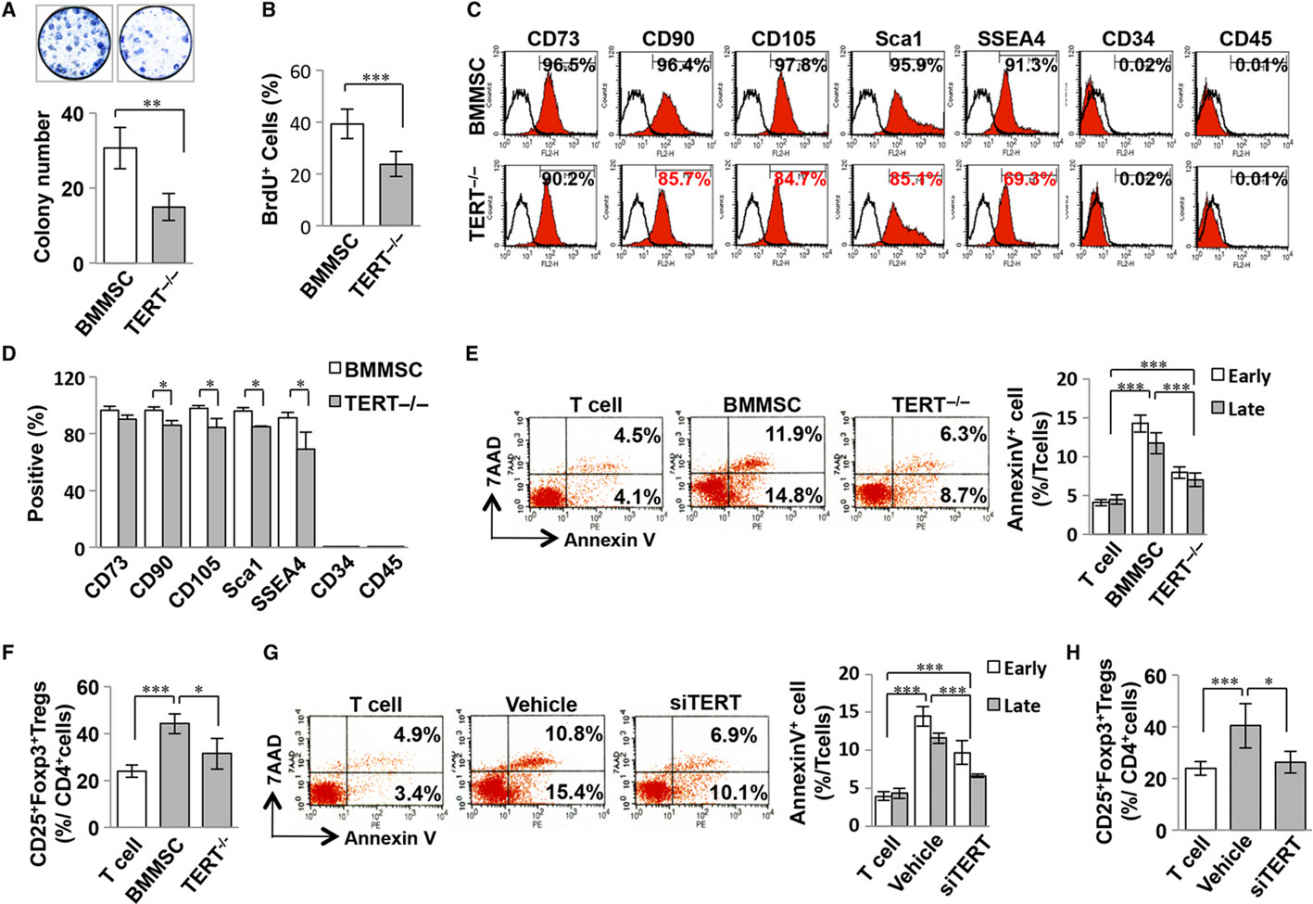
Telomerase reverse transcriptase (TERT) contributes to Bone marrow mesenchymal stem cell (BMMSC)-mediated immunomodulation. A *TERT*^−*/*−^ BMMSCs showed reduced single colony-forming ability. (Student's *t*-test, *n* = 5 in each group, **P <* *0.01). Error bars present the s.d. of the mean values. B BrdU labeling was performed to show reduced proliferative capacity of *TERT*^−*/*−^ BMMSCs (Student's *t*-test, *n* = 5 in each group, ***P <* *0.005). Error bars present the s.d. of the mean values. C–D FACS analysis showed downregulated expression levels of BMMSC surface markers in *TERT*^−*/*−^ BMMSCs (Student's *t*-test, *n* = 5 in each group, *P <* *0.005). Error bars present the s.d. of the mean values. E *In vitro* coculture system showed a significantly decreased capacity of *TERT*^−*/*−^ BMMSCs to induce AnnexinV^+^7AAD^−^ and AnnexinV^+^7AAD^+^ double positive apoptotic T cells compared to regular (WT) BMMSCs (One-way ANOVA, Bonferroni, *n* = 5 in each group, ***P <* *0.005). Error bars present the s.d. of the mean values. F Treg induction assay indicated a significantly decreased capacity of *TERT*^−*/*−^ BMMSCs to upregulate Treg levels in comparison with WT BMMSCs (One-way ANOVA, Bonferroni, *n* = 5 in each group, ***P <* *0.005, *P <* *0.05). Error bars present the s.d. of the mean values. G *In vitro* coculture system showed a decreased capacity of *TERT* knockdown BMMSCs by siRNA to induce AnnexinV^+^7AAD^−^ and AnnexinV^+^7AAD^+^ double positive apoptotic T cells BMMSCs (One-way ANOVA, Bonferroni, *n* = 5 in each group, ***P <* *0.005). Error bars present the s.d. of the mean values. H Treg induction assay was performed to show a significantly decreased capacity of *TERT* knockdown BMMSCs to upregulate the level of Tregs in comparison with WT BMMSCs (One-way ANOVA, Bonferroni, *n* = 5 in each group, ***P <* *0.005, *P <* *0.05). Error bars present the s.d. of the mean values. Vehicle: scrambled siRNA-treated BMMSCs.

To address whether TERT is important in regulating BMMSC-mediated immunomodulation, we isolated BMMSCs from TERT null mice, B6.129S-*Tert*^*tm1Yjc*^/J (*TERT*^−*/*−^), and found that the number of single colony clusters (colony-forming unit fibroblasts, CFU-F) was significantly reduced in *TERT*^−*/*−^ BMMSCs (Fig 1A). To examine the proliferative capacity of *TERT*^−*/*−^ BMMSCs, we performed a BrdU-labeling assay to show that *TERT*^−*/*−^ BMMSCs have a reduced proliferative rate compared to *TERT*^*+/+*^ age-matched littermate (wild-type) BMMSCs (Fig 1B). Flow cytometric analysis showed generally lower expression of mesenchymal stem cell surface molecules, including CD90, CD105, Sca1, and SSEA4, in *TERT*^−*/*−^ BMMSCs, while the hematopoietic lineage markers CD34 and CD45 were absent in *TERT*^−*/*−^ BMMSCs, similar to observations of BMMSCs derived from wild type mice (WT BMMSCs) (Fig 1C and D). We observed that *TERT*^−*/*−^ BMMSCs exhibited decreased osteogenic differentiation and increased adipogenic differentiation potential, as indicated by alizarin red staining of mineralized nodule formation and Oil red O staining of lipid-containing adipocytes, respectively. As expected, gene expression analysis revealed downregulated expression of the osteogenic genes runt-related transcription factor 2 (*runx2*) and osteocalcin (*ocn*), along with upregulated expression of the adipogenic genes peroxisome proliferator-activated receptor gamma 2 (*ppar*γ*2*) and lipoprotein lipase (*lpl*) in *TERT*^−*/*−^ BMMSCs (supplementary Fig S1E–H).

Next, we used a BMMSC/T-cell co-culture system to show that *TERT*^−*/*−^ BMMSCs had significantly reduced capacity to induce AnnexinV^+^7AAD^−^ and AnnexinV^+^7AAD^+^ double positive apoptotic T cells and upregulate CD4^+^CD25^+^Foxp3^+^ regulatory T cells (Tregs), when compared to the WT BMMSCs (Figs 1E and F). Western blot analysis confirmed the absence of telomerase activity and TERT expression in *TERT*^−*/*−^ BMMSCs, as assessed by a telomeric repeat amplification protocol (TRAP)-ELISA assay and Western blot, respectively (supplementary Fig S1A and B). In addition, we performed quantitative PCR (qPCR) to examine the RNA level of *TERT* from passage-0 to passage-10 to further confirm our Western blot data. *TERT* expression is maintained at a certain level from P0 to P2 of WT BMMSCs, which were used in this study. However, the expression level of *TERT* was significantly decreased in passage 5 and undetectable by qPCR in passage 10. On the other hand, *TERT* expression was undetectable in *TERT*^−*/*−^ BMMSCs from p0 to p10 (supplementary Fig S1A). Moreover, siRNA-mediated knockdown of *TERT* expression in BMMSCs showed that TERT expression levels and telomerase activity were markedly decreased in *TERT* knockdown BMMSCs compared to the scrambled siRNA treated BMMSCs (supplementary Fig S1C and D). *TERT* knockdown BMMSCs also showed a significantly decreased capacity to induce T-cell apoptosis and upregulate Tregs when compared to the WT BMMSCs (Fig 1G and H). Previous studies have reported that aged BMMSCs exhibit decreased proliferation and differentiation potential (Bonab *et al*, 2006). We found that BMMSCs from 6-month-old mice (6M-BMMSCs) had downregulated levels of TERT and a reduced capacity to induce T-cell apoptosis and upregulate Tregs when compared to BMMSCs from 1-month-old mice (supplementary Fig S2A–C). Thus, we have used *TERT*^−*/*−^, *TERT* knockdown and BMMSCs from mice of different ages to demonstrate the key role telomerase plays in governing BMMSC-based immunomodulation.

### TERT is required for BMMSC-mediated amelioration of disease phenotype in systemic sclerosis mice

**Figure 2:**
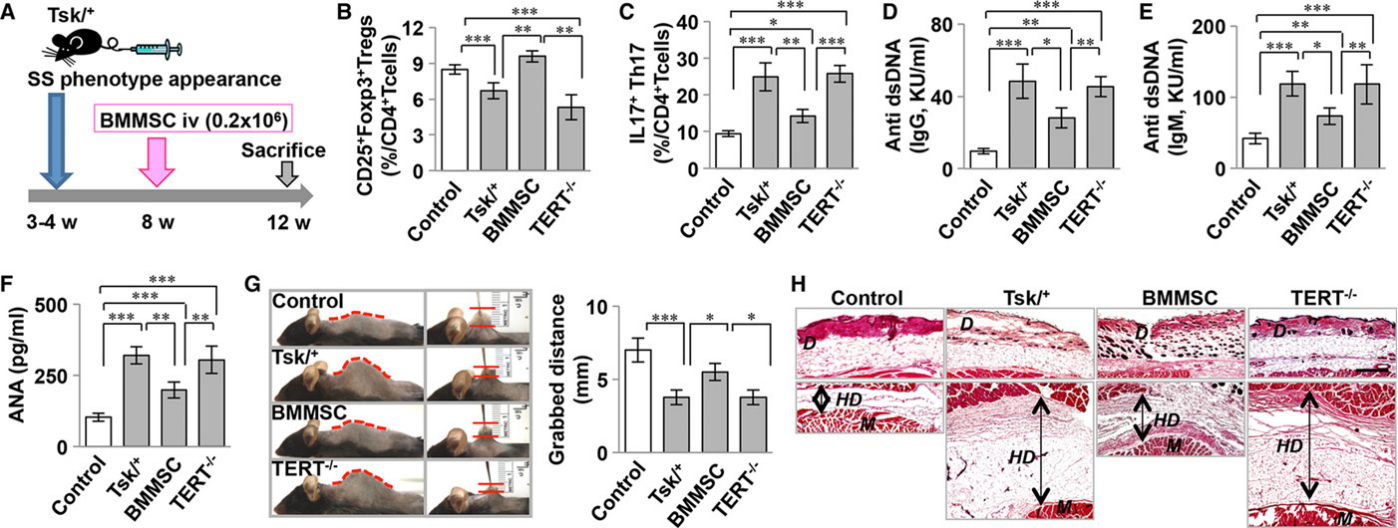
Telomerase reverse transcriptase (TERT) is required for Bone marrow mesenchymal stem cell (BMMSC)-mediated amelioration of disease phenotype in systemic sclerosis mice. A Schema showing BMMSC transplantation (MSCT) for treating systemic sclerosis (SS) tight skin (Tsk/^+^) mice. B FACS analysis showed that the Treg level was significantly decreased in Tsk/^+^ mice compared to *Fbn1*^*+/+*^ control littermates. After MSCT, the Treg level was significantly elevated, whereas *TERT*^−*/*−^ MSCT failed to upregulate the Treg level in Tsk/^+^ mice. C FACS analysis showed that CD4^+^IL17^+^ Th17 cells were significantly increased in Tsk/^+^ mice compared to control littermates. WT MSCT, but not *TERT*^−*/*−^ MSCT, was able to significantly reduce the Th17 level in Tsk/^+^ mice. D–F ELISA assays showed that Tsk/^+^ mice had elevated levels of anti-double strand DNA antibodies IgG (D), IgM (E) and antinuclear antibody (ANA, F) when compared to control littermates. WT MSCT reduced the levels of anti-double strand DNA antibodies IgG (d), IgM (e) and ANA (f). In contrast, *TERT*^−*/*−^ MSCT failed to reduce the levels of anti-double strand DNA antibodies IgG (d), IgM (e) and ANA (f). G Tsk/^+^ mice showed a tight skin phenotype. Grabbed skin distance measurement showed that WT BMMSC, but not *TERT*^−*/*−^ BMMSC, transplantation significantly improved the tight skin phenotype. H Histological examination identified that hyperdermal thickness was significantly increased in Tsk/^+^ mice compared to control littermates. WT BMMSC, but not *TERT*^−*/*−^ BMMSC, transplantation improved hyperdermal thickness in Tsk/^+^ mice. Scale Bar, 100 μm. *D*: Dermal, *M*: Muscle, and HD: Hyperdermal. Data information: Error bars represent the s.d. from the mean values (One-way ANOVA, Bonferroni, *n* = 6 in each group, ***P <* *0.005, **P <* *0.01, *P <* *0.05).

Recently, immunomodulatory properties were identified as an important characteristic of BMMSCs, which has led to their systemic infusion to treat a variety of immune diseases (Aggarwal ' Pittenger, 2005; Nauta ' Fibbe, 2007; Uccelli *et al*, 2007, 2008). Therefore, in order to assess the therapeutic effect of *TERT*^−*/*−^ BMMSCs, we infused either regular BMMSCs (WT; from *TERT*^*+/+*^ littermates) or *TERT*^−*/*−^ BMMSCs into B6.Cg-*Fbn1*^*Tsk*^/J (Tsk/^+^) systemic sclerosis (SS) mice at 8 weeks of age and analyzed treatment response at 12 weeks of age (Fig 2A). Flow cytometric analysis revealed that WT BMMSC transplantation (MSCT) significantly upregulated the number of Tregs and downregulated the number of CD4^+^IL17^+^IFNγ^−^ T helper 17 (Th17) cells in comparison to the untreated group, while *TERT*^−*/*−^ MSCT failed to either upregulate Treg levels or downregulate the level of Th17 cells in SS mice (Fig 2B and C). Furthermore, SS mice showed a significant increase in the levels of antinuclear antibody (ANA) and anti-double strand DNA (dsDNA) IgG and IgM antibodies in serum. WT MSCT, but not *TERT*^−*/*−^ MSCT, showed significant reduction in the levels of ANA, and dsDNA IgG and IgM in SS mice (Fig 2D–F). Additionally, the tightness of skin, as measured by grabbed distance, was significantly improved in the WT MSCT group, but not the *TERT*^−*/*−^ MSCT group (Fig 2G). Histological analysis also confirmed that skin hypodermal thickness was significantly increased in SS mice. After WT MSCT, hypodermal thickness was reduced to a level equal to that of the control group, whereas *TERT*^−*/*−^ MSCT failed to reduce hypodermal thickness (Fig 2H). These data indicate that *TERT*^−*/*−^ MSCT failed to offer effective treatment for SS mice.

### TERT promotes FASL expression in BMMSCs through Wnt/β-catenin pathway

**Figure 3:**
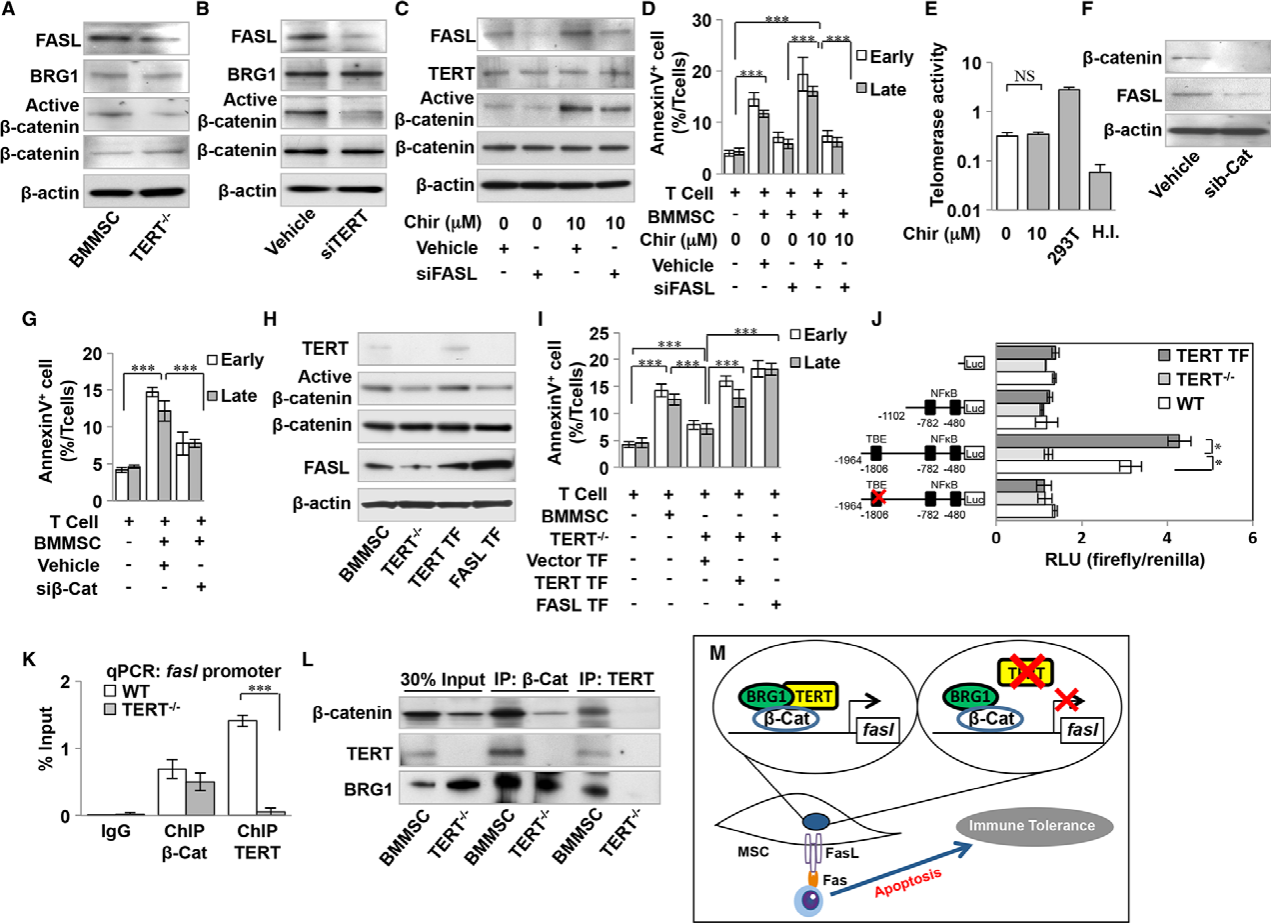
Telomerase reverse transcriptase (TERT) serves as a transcriptional modulator to regulate FASL expression in Bone marrow mesenchymal stem cells (BMMSCs). A–B Western blot analysis showed decreased levels of FASL and active β-catenin, but not BRG1, in *TERT*^−*/*−^ BMMSCs (A) and *tert* knockdown BMMSCs by siRNA (B) compared to *TERT*^*+/+*^ (WT) BMMSCs. C β-catenin activator (Chir, 10 μM) treatment elevated levels of active β-catenin and FASL in WT BMMSCs. *fasl* knockdown BMMSCs by siRNA showed a decreased level of FASL expression, but not active β-catenin. D *In vitro* coculture system showed β-catenin activator (Chir)-treated BMMSCs had increased capacity to induce AnnexinV^+^7AAD^−^ and AnnexinV^+^7AAD^+^ double positive apoptotic T cells compared to control group. *fasl* siRNA treatment could reduce Chir-elevated T cell apoptosis in the co-culture system. E Telomerase activity in Chir-treated BMMSCs showed no significant difference from the untreated group. 293T cells were used as a positive control, and heat-inactivated (H.I.) samples were used as a negative control. F Western blot analysis showed decreased expression levels of β-catenin and FASL in β-catenin knockdown BMMSCs by siRNA. G β-catenin knockdown BMMSCs by siRNA showed decreased capacity to induce AnnexinV^+^7AAD^−^ and AnnexinV^+^7AAD^+^ double positive apoptotic T cells compared to the control siRNA group. H Western blot showed that *TERT*^−*/*−^ BMMSCs decreased expression levels of TERT, active β-catenin, and FASL. *Tert* transfection (TERT TF) rescued the expression levels of TERT, active β-catenin, and FASL, assessed by Western blot, while *fasl* transfection (FASL TF) only rescued FASL expression, but not that of TERT or β-catenin, in *TERT*^−*/*−^ BMMSCs. I *In vitro* coculture system showed a decreased capacity of *TERT*^−*/*−^ BMMSCs to induce AnnexinV^+^7AAD^−^ and AnnexinV^+^7AAD^+^ double positive apoptotic T cells when compared to the control group, whereas transfection of both *tert* and *fasl* rescued the capacity to induce AnnexinV^+^7AAD^−^ and AnnexinV^+^7AAD^+^ double positive apoptotic T cells. J *fasl* promoter-luciferase fusions were examined in WT, *TERT*^−*/*−^ and TERT TF BMMSCs. Promoter activity was expressed as relative light units (RLU) normalized to the activity of co-transfected Renilla luciferase. The activity of 2 kb promoter-luciferase fusion was significantly elevated compared to 1.1 kb fusion in WT BMMSCs and TERT TF BMMSCs when compared to *TERT*^−*/*−^ BMMSCs. The activity of TBE-specific site-mutated promoters was markedly decreased in WT BMMSCs and TERT TF BMMSCs. K Chromatin immunoprecipitation (ChIP)-qPCR assay showed enrichment of direct association of β-catenin on the *fasl* promoter in WT and *TERT*^−*/*−^ BMMSCs, while the enrichment of direct association of TERT on the *fasl* promoter was only found in WT BMMSCs. L ChIP-Western blot assays showed direct association of TERT, β-catenin and BRG1 on the *fasl* promoter in WT BMMSCs, but only direct association of β-catenin and BRG1 on the *fasl* promoter in *TERT*^−*/*−^ BMMSCs. M Schematic diagram indicates that TERT, as a transcriptional modulator in a complex with β-catenin and BRG1, mediates FASL expression in BMMSC-induced immunoregulation. Vehicle: scrambled siRNA-treated BMMSCs. Data information: Error bars represent the s.d. from the mean values (One-way ANOVA, Bonferroni, *n* = 3 in each group, ***P <* *0.005, *P <* *0.05). Source data are available for this figure.

In order to learn how telomerase activity contributes to BMMSC-mediated immunomodulation, we examined the levels of BMMSC-associated immunomodulatory factors, including IL-10, PGE2, and FASL, in *TERT*^−*/*−^ and TERT knockdown BMMSCs. ELISA analysis showed that IL-10 and PGE2 were not significantly altered in either *TERT* null or knockdown BMMSCs (supplementary Fig S3A and B). However, Western blot analysis indicated that the FASL expression level was markedly decreased in both *TERT* null and knockdown BMMSCs (Fig 3A and B). To further confirm that FASL is required for BMMSC-mediated immunosuppression, we isolated *FASL* mutant BMMSCs from B6Smn.C3-*Fasl*^*gld*^/J mice (*gld*BMMSC) and examined their immunomodulatory properties in an *in vitro* coculture system. The capacity of *gld*BMMSCs to induce T-cell apoptosis was significantly decreased when compared to WT BMMSCs (supplementary Fig S3C). These findings are supported by previous studies which showed that FASL plays a crucial role in BMMSC-based immunomodulation (Akiyama *et al*, 2012). In the present study, we isolated FASL^+^ and FASL^−^ subpopulations of BMMSCs by fluorescence cell sorting (supplementary Fig S4A) and used a T-cell co-culture system to show that FASL^+^ BMMSCs had an increased capacity to induce both AnnexinV^+^7AAD^−^ and AnnexinV^+^7AAD^+^ double positive apoptotic T cells when compared to WT BMMSCs, while FASL^−^ BMMSCs lost this immunomodulatory function in the *in vitro* co-culture system, confirming that FASL expression affects the immunomodulatory properties of BMMSCs (supplementary Fig S4B).

It has been reported that TERT is able to act as a cofactor to modulate transcriptional responses by regulating the Wnt signaling pathway and is also able to execute a stem cell activation program by interacting with the chromatin-remodeling protein BRG1 (Park *et al*, 2009). We next examined the expression levels of Wnt/β-catenin and BRG1 in *TERT*-deficient BMMSCs. Western blot analysis showed that the expression level of active β-catenin (non-phosphorylated), but not BRG1, was markedly decreased in both *TERT* null and knockdown BMMSCs (Fig 3A and B). β-catenin activator (CHIRON 99021) treatment could significantly elevate expression levels of activated β-catenin and FASL, but not TERT, in BMMSCs. FASL knockdown by siRNA in β-catenin activator-treated BMMSCs significantly diminished the FASL expression level, but not that of TERT or activated β-catenin (Fig 3C). Co-culture of BMMSCs and T cells indicated that β-catenin activator treatment could significantly elevate the capacity of BMMSCs to induce both AnnexinV^+^7AAD^−^ and AnnexinV^+^7AAD^+^ double positive apoptotic T cells when compared to the untreated group, but that such elevation could be abrogated by FASL siRNA treatment (Fig 3D). TRAP-ELISA assays also showed that β-catenin activator treatment failed to affect telomerase activity when compared to WT BMMSCs (Fig 3E). Downregulation of β-catenin expression in BMMSCs resulted in decreased FASL expression levels, as evaluated by Western blot assay, confirming that Wnt/β-catenin signaling regulated FASL (Fig 3F). Flow cytometric analysis showed that β-catenin knockdown BMMSCs have a reduced capacity to induce AnnexinV^+^7AAD^−^ and AnnexinV^+^7AAD^+^ double positive apoptotic T cells (Fig 3G). Interestingly, overexpression of *tert* in *TERT*^−*/*−^ BMMSCs (TERT TF) rescued the expression levels of TERT, active β-catenin, and FASL, as well as the capacity to induce T-cell apoptosis, while *fasl* overexpression (FASL TF) only elevated FASL expression, but also rescued the capacity to induce T-cell apoptosis (Fig 3H and I). This experimental evidence suggests that TERT serves as an upstream activator of Wnt/β-catenin signaling to regulate FASL expression, which, in turn, regulates the immunomodulatory properties of BMMSCs.

### TERT serves as a transcriptional modulator to regulate FASL expression in BMMSCs

To examine whether β-catenin directly controls FASL expression at the transcriptional level, we used PROMO search tools (http://alggen.lsi.upc.es/) to examine the *fasl* promoter sequence. We found two possible transcription factor candidate binding sites, TCF/LEF1 binding element (TBE) and nuclear factor kappa B (NFκB), both closely matching the consensus targets. We therefore generated 1.1 kb (only NFkB targets) and 2 kb (both TBE and NFκB targets) *fasl* promoter reporter constructs in which the defined region of the *fasl* promoter and flanking region were placed upstream of a reporter gene encoding firefly luciferase (Fig 3J). Luciferase reporter analysis demonstrated that the 2 kb construct, but not the 1.1 kb construct, showed markedly higher promoter activity in both normal BMMSCs (WT) and *tert* overexpressed BMMSCs (TERT TF) compared to TERT null BMMSCs (*TERT*^−/−^), suggesting that the TBE transcriptional element may contribute to FASL expression. When *TERT*^−*/*−^ BMMSCs were transfected with a reporter vector, the luciferase assay showed significantly decreased promoter activity. Introduction of a TBE-mutated reporter vector markedly diminished the expression of the *fasl*-luciferase reporter, suggesting a direct initiation of *fasl* expression by Wnt/β-catenin cascades (Fig 3J). We next determined whether β-catenin directly binds to the *fasl* promoter in BMMSCs. Using chromatin immunoprecipitation (ChIP)-qPCR, the TBE binding consensus sequence within the promoter region was examined to determine its ability to recruit β-catenin. Unexpectedly, the β-catenin-bound DNA at the candidate site was significantly enriched in both normal and *TERT*^−*/*−^ BMMSCs (Fig 3K). These findings prompted us to examine whether TERT contributes to this transcriptional process. ChIP-qPCR analysis demonstrated that TERT-bound DNA at the candidate site was enriched in normal BMMSCs, but not *TERT*^−*/*−^ BMMSCs, indicating that TERT acts as a cofactor with β-catenin to drive FASL expression (Fig 3K). To further confirm the role of TERT and β-catenin in binding to the *fasl* promoter, immunoprecipitation of nuclear protein by either β-catenin or TERT antibodies was performed. The results showed that TERT, β-catenin, and BRG1 formed a complex in normal BMMSCs, while only β-catenin and BRG1 formed a complex in *TERT*^−*/*−^ BMMSCs (Fig 3L). Together, these findings indicate that TERT, together with β-catenin, serves as a transcriptional regulator of FASL expression (Fig 3M).

### Aspirin pretreatment increases immunomodulation of BMMSCs through TERT activation

**Figure 4:**
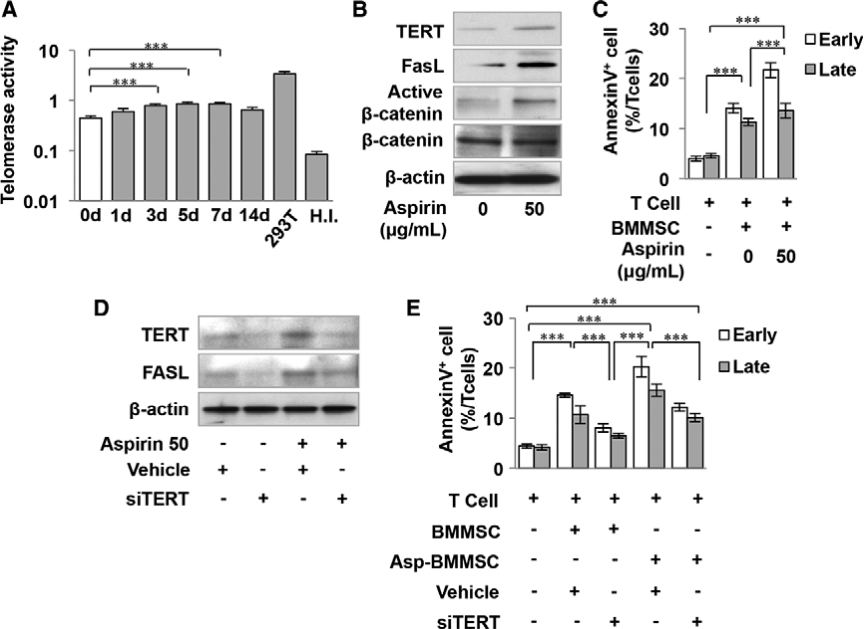
Aspirin pretreatment increases immunomodulation of Bone marrow mesenchymal stem cells (BMMSCs) through Telomerase reverse transcriptase (TERT) activation.
TRAP-ELISA assays showed that Aspirin-pretreated (50 μg/ml) BMMSCs exhibited increased telomerase activity from day 3 to day 7 compared to the untreated group. 293T cells were used as a positive control, and heat-inactivated (H.I.) samples were used as negative control.Western blot analysis showed elevated levels of TERT, FASL, and active β-catenin expression in aspirin-pretreated BMMSCs.*In vitro* coculture system showed that Aspirin-pretreated BMMSCs exhibited increased capacity to induce AnnexinV^+^7AAD^−^ and AnnexinV^+^7AAD^+^ double positive apoptotic T cells compared to the untreated group.Western blot analysis showed that aspirin pretreatment elevated levels of TERT and FASL expression in BMMSCs, which could be blocked by *TERT* siRNA treatment.*In vitro* coculture system showed that aspirin-pretreated BMMSCs exhibited increased capacity to induce AnnexinV^+^7AAD^−^ and AnnexinV^+^7AAD^+^ double positive apoptotic T cells, which could be diminished by *TERT* siRNA treatment. Vehicle: scrambled siRNA-treated BMMSCs. Data information: Error bars represent the s.d. from the mean values (One-way ANOVA, Bonferroni, *n* = 3 in each group, ***P <* *0.005). Source data are available for this figure.

Previously, we reported that aspirin-pretreated BMMSCs (Asp-BMMSCs) showed increased telomerase activity and improved bone regeneration (Yamaza *et al*, 2008). Thus, we examined whether aspirin-induced telomerase activity could improve BMMSC-based immunoregulation. A TRAP-ELISA assay showed that telomerase activity was significantly increased at 3 days after aspirin pretreatment and that it maintained this elevated level for more than 7 days (Fig 4A). To examine whether upregulated TERT activity by aspirin is specific to BMMSCs, naïve T cells from splenocytes were isolated and pretreated with aspirin for 3 days. TRAP-ELISA assays showed that telomerase activity was elevated in the BMMSC group, but not in the naïve T-cell group (supplementary Fig S5A). We found that Asp-BMMSCs expressed higher levels of TERT, active β-catenin, and FASL and showed elevated capacity to induce AnnexinV^+^7AAD^−^ and AnnexinV^+^7AAD^+^ double positive apoptotic T cells when compared to the untreated group (Fig 4B and C). Knockdown TERT expression in Asp-BMMSCs by siRNA significantly decreased the expression levels of TERT and FASL and the capacity to induce T-cell apoptosis, suggesting that aspirin elevated the immunomodulatory capacity of BMMSCs through TERT activation (Fig 4D and E). These data suggest that aspirin pretreatment increases telomerase activity and elevates the immunomodulation capacity of mouse BMMSCs. To further extend these findings to clinical application, human BMMSCs were isolated and pretreated with aspirin. We verified that Asp-BMMSCs from human bone marrow showed increased telomerase activity using a TRAP-ELISA assay; they also showed increased TERT expression levels, as evaluated by Western blot analysis, when compared to the non-pretreated group (supplementary Fig S5B). To confirm that elevated telomerase activity was related to the immunomodulatory properties of Asp-BMMSCs, BMMSC-T-cell co-culture experiments were performed to show that Asp-BMMSCs induced increased T-cell apoptosis when compared to the BMMSC group (supplementary Fig S5B). In addition, cytogenetic analysis of Asp-BMMSCs showed no karyotype alterations, suggesting that *in vitro* aspirin pretreatment (50 μg/ml) may be a safe approach to improve BMMSC immunomodulatory properties (supplementary Fig S5C).

### Aspirin-pretreated BMMSCs show increased capacity to ameliorate systemic sclerosis phenotypes

**Figure 5:**
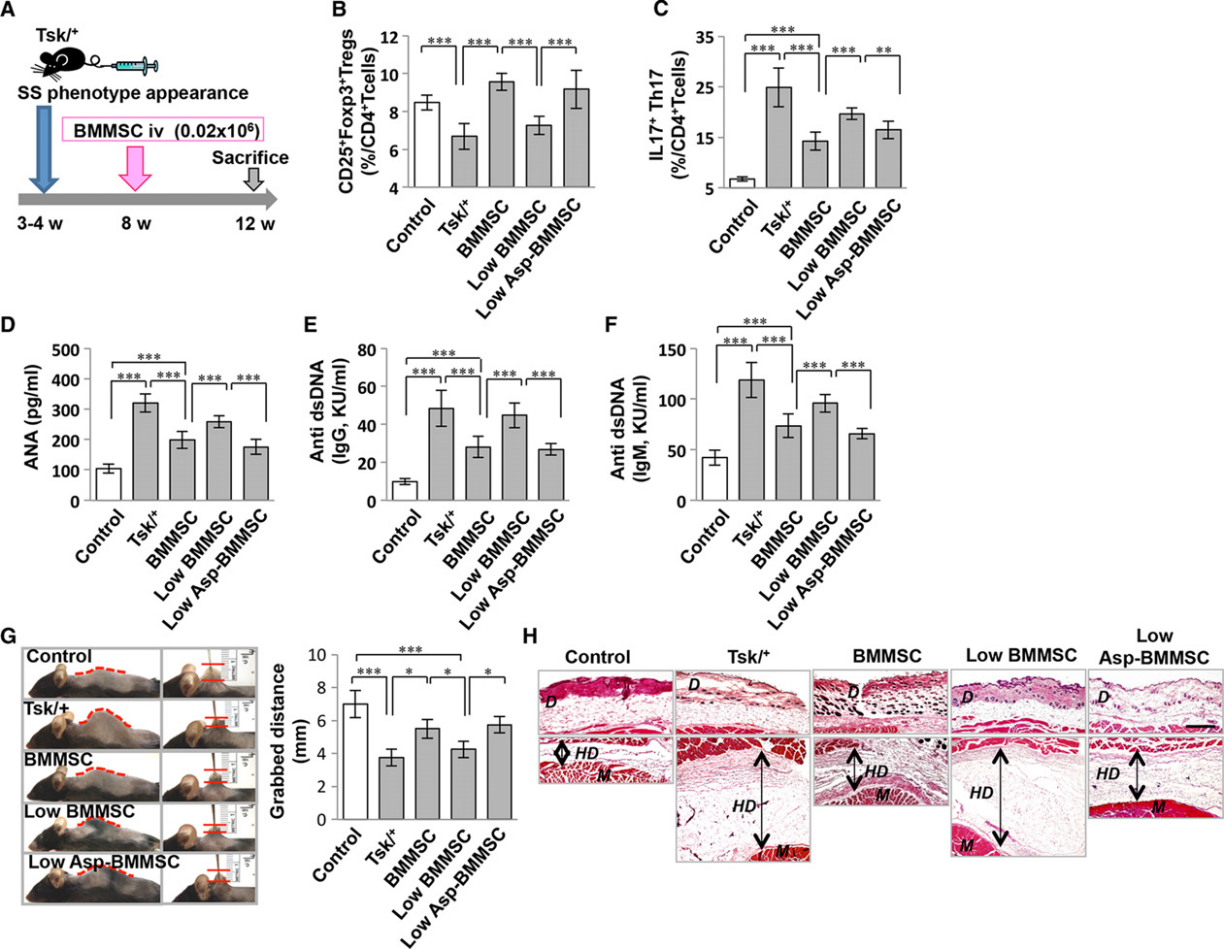
Aspirin-pretreated BMMSCs show increased capacity to ameliorate systemic sclerosis phenotypes.. A Schema showing procedure for using aspirin-pretreated BMMSC transplantation (Asp-MSCT) to treat systemic sclerosis (SS) tight skin (Tsk/^+^) mice. B FACS analysis showed that the Treg level was significantly decreased in Tsk/^+^ mice compared to control littermates. After MSCT, the Treg level was significantly elevated. Using ten times less BMMSCs during MSCT (Low-BMMSC) failed to increase the Treg level, while Low-Asp-BMMSC significantly elevated the Treg level in Tsk/^+^ mice. C FACS analysis showed that CD4^+^IL17^+^ Th17 cells were significantly increased in Tsk/^+^ mice compared to control littermates. After MSCT, the Th17 level was significantly reduced. Low-BMMSC failed to diminish Th17 level, while Low-Asp-BMMSC significantly reduced the Th17 level in Tsk/^+^ mice. D–F ELISA assays showed that Tsk/^+^ mice had elevated levels of antinuclear antibody (ANA, D) and anti-double strand DNA antibodies IgG (E) and IgM (F) when compared to control littermates. MSCT reduced the levels of ANA (d) and anti-double strand DNA antibodies IgG (e) and IgM (f). Low-BMMSC failed to diminish the levels of ANA (D) and anti-double strand DNA antibodies IgG (E) and IgM (F), while Low-Asp-BMMSC significantly reduced the levels of ANA (D) and anti-double strand DNA antibodies IgG (E) and IgM (F) in Tsk/^+^ mice. G Tsk/^+^ mice showed tight skin phenotype compared to control littermates. MSCT significantly improved skin phenotype in terms of grabbed skin distance in Tsk/^+^ mice. Grabbed skin distance measurement showed that Low-BMMSC failed to ameliorate tight skin phenotypes, while Low-Asp-BMMSC significantly improved skin phenotypes in Tsk/^+^ mice. H Hyperdermal thickness was significantly increased in Tsk/^+^ mice compared to control littermates. MSCT improved hyperdermal thickness in Tsk/^+^ mice. Histological examination identified that Low-BMMSC failed to diminish hyperdermal thickness, while Low-Asp-BMMSC significantly reduced hyperdermal thickness in Tsk/^+^ mice. Scale Bar, 100 μm. *D*: Dermal, *M*: Muscle, and HD: Hyperdermal. Data information: Error bars represent the s.d. from the mean values (One-way ANOVA, Bonferroni, *n* = 6 in each group, ***P <* *0.005, **P <* *0.01, *P <* *0.05).

Treatment with 0.2 × 10^6^ BMMSCs (positive control group in our study) is considered a standard dosage to elicit a therapeutic response. Therefore, we infused 10% of that amount (0.02 × 10^6^ of either Asp-BMMSCs or BMMSCs) into Tsk/^+^ SS mice at 8 weeks of age to examine whether aspirin pretreatment could reduce the dosage of BMMSCs in immunotherapy (Fig 5A). When we analyzed treatment responses at 12 weeks of age, we found that a 10% dose of Asp-BMMSCs, but not 10% untreated BMMSCs, was capable of elevating Treg levels equal to that of the positive control group (Fig 5B). Flow cytometric analysis further revealed that the 10% Asp-BMMSCs group showed a greater efficacy in reducing the number of Th17 cells, levels of ANA and dsDNA IgG and IgM antibodies in peripheral blood, when compared to the 10% untreated BMMSCs group (Fig 5C–F). In addition, the tightness of skin, as measured by grabbed distance, was significantly improved in the 10% Asp-BMMSCs group, but not the 10% BMMSCs group in Tsk/^+^ mice (Fig 5G). Histological analysis also confirmed that skin hypodermal thickness was significantly reduced to a level equal to that of the control group in the 10% dose of Asp-BMMSCs group, whereas the 10% dose of untreated BMMSCs group failed to reduce hypodermal thickness (Fig 5H). Moreover, the 10% Asp-BMMSCs group showed a therapeutic effect similar to that observed in the positive control. To confirm that aspirin-elevated telomerase activity contributes to BMMSC-mediated immune therapy, we infused *TERT*^−*/*−^ BMMSCs, with or without aspirin pretreatment, into Tsk/^+^ mice and found that they failed to rescue the disease phenotypes, as indicated by no significant changes in terms of the levels of Tregs, Th17, ANA, and dsDNA IgG and IgM antibodies in peripheral blood, tightness of skin, and histological skin hypodermal thickness when compared to the untreated WT BMMSC infusion group (supplementary Fig S6A–H). In addition, infusion of aspirin alone also failed to rescue the disease phenotypes (supplementary Fig S6A–H). These data lead us to hypothesize that the number of BMMSCs used for immunotherapy could be dramatically reduced if BMMSCs are first treated with a telomerase activator, such as aspirin.

## Discussion

Bone marrow mesenchymal stem cells exhibit immunomodulatory functions by mediating the proliferation, migration and function of several major types of immune cells, and systemic infusion of BMMSCs has been shown to yield therapeutic benefits for a variety of immune disorders (Le Blanc *et al*, 2004; Aggarwal ' Pittenger, 2005; Chen *et al*, 2006; Nauta ' Fibbe, 2007; Uccelli *et al*, 2007, 2008; Liang *et al*, 2009, 2012; Sun *et al*, 2009; Scuderi *et al*, 2013; Liu *et al*, 2013). However, the stem cell properties of BMMSCs in maintaining immunomodulatory function are poorly understood. Specifically, it is yet to be determined whether the unique gene-driven functional commonality of stem cells, including BMMSCs, plays a role in regulating immune response at therapeutic levels. Previous reports have demonstrated that telomerase reverse transcriptase (TERT) plays a key role in progenitor cell survival and stem cell self-renewal and that it controls telomere maintenance to ensure chromosome stability (Maser ' DePinho, 2002; Shi *et al*, 2002; Gronthos *et al*, 2003; Smogorzewska ' de Lange, 2004; Yamaza *et al*, 2008; Liu *et al*, 2011). In the present study, we found that expression of MSC surface markers, including CD90, CD105, Sca1 and SSEA4, was significantly reduced in *TERT*^−*/*−^ BMMSCs. CD90 may function as an activator for stem cell differentiation (Chen *et al*, 1999). CD105 is important for BMMSC adhesion and angiogenesis (Duff *et al*, 2003). Sca1 and SSEA4 are general stem cell markers for BMMSCs (Gang *et al*, 2007; Battula *et al*, 2009). TERT is highly expressed in newly isolated BMMSCs from aspirates of bone marrow (Gronthos *et al*, 2003) and is rapidly downregulated in BMMSCs during *ex vivo* expansion (Shi *et al*, 2002). TERT may contribute to the maintenance of stem cell properties of BMMSCs.

Clinically, a correlation between successful immunosuppressive therapy and the dosage of donor stem cells has been well documented (Liang *et al*, 2010; Wang *et al*, 2012). In this study, we reveal a novel mechanism by which telomerase governs the immunomodulatory properties of BMMSCs via upregulation of the FASL-induced apoptotic pathway. Further, activation of telomerase activity in BMMSCs by a pharmacological approach, such as *in vitro* aspirin treatment, can markedly improve BMMSC-based immunomodulation and reduce the number of BMMSCs used for immunotherapy in SS mice. By a mechanistic study, we reveal that telomerase-enhanced FASL production is associated with Wnt/β-catenin signaling, in which a TERT/β-catenin/BRG1 complex directly binds to the *fasl* promoter to drive gene expression at the transcriptional level. This study provides experimental evidence that links telomerase activity to BMMSC-based immunomodulation and demonstrates the potential to improve BMMSC-based clinical therapies with reduced cell dosage.

It has been reported that aspirin promotes osteogenesis and bone regeneration (Yamaza *et al*, 2008; Liu *et al*, 2011), but inhibits proliferation (Wang *et al*, 2006; Deng *et al*, 2009) in BMMSCs. Activation of Wnt/β-catenin by aspirin treatment may contribute to elevated osteogenesis (Yamaza *et al*, 2008); however, the detailed mechanism is not clear. Moreover, it is well known that TERT can regulate the Wnt/β-catenin pathway (Choi *et al*, 2008; Park *et al*, 2009). In this study, we showed that TERT associates with β-catenin to form a transcriptional complex to control the expression of FASL, thereby affecting BMMSC-mediated immunomodulation. Aspirin is a widely used anti-inflammatory drug, and treatment using a 50 μg/ml dosage appears to have no negative effect on BMMSCs. It is therefore reasonable to continue examining the efficacy of using aspirin-treated BMMSCs for clinical therapies.

Taken together, this translational study substantially extends current knowledge about BMMSC-based immunotherapy and provides a new strategy for improving it. We also reveal a novel mechanism by which TERT is, for the first time, linked to BMMSC-mediated immunomodulation.

## Materials and Methods

### Animals

C57BL/6J, B6.129S-*Tert*^*tm1Yjc*^/J (*TERT*^−*/*−^), B6.Cg-*Fbn1*^*Tsk*^/J (Tsk/^+^) and B6Smn.C3-*Fasl*^*gld*^/J mouse lines were purchased from the Jackson Lab. To maintain the *TERT*^−*/*−^ strain and generate *TERT*^*+/+*^ (WT) mice, heterozygous (*TERT*^*+/*−^) pairs were intercrossed. Also, *TERT*^−*/*−^ mice were intercrossed to produce telomerase deficient fourth generation (G4) mice. Due to the embryonic lethality of a homozygous *Fbn1* mutation, heterozygous Tsk/^+^ mice were intercrossed to generate WT and heterozygous Tsk/^+^ mice for the SS disease model. Aged-matched female littermates were used as controls in the present study. All animal experiments were performed under institutionally approved protocols for the use of animal research (USC #11141).

### Antibodies

Anti-SSEA4, active β-catenin and total β-catenin monoclonal antibodies were purchased from Millipore (Billerica, MA, USA). Anti-Sca1-PE, CD34-PE, CD45-PE, CD73-PE, CD4-PerCP, CD8-FITC, CD25-APC, CD3ε and CD28 antibodies were purchased from BD Biosciences (San Jose, CA, USA). Anti-CD105-PE, CD178(FASL)-PE, Foxp3-PE, IL17-PE, and IFNγ-APC antibodies were purchased from eBioscience (San Diego, CA, USA). Anti-TERT, FASL and total β-catenin (ChIP grade) polyclonal antibodies were purchased from Santa Cruz Biotechnology (Dallas, TX, USA). Anti-BRG1 antibody was purchased from Cell Signaling (Danvers, MA, USA). Anti-β-Actin antibody was purchased from Sigma-Aldrich (St. Louis, MO, USA).

### Isolation and culture of mouse BMMSCs

Single cell suspension was obtained from bone marrow-derived all nuclear cells (ANCs) taken from femurs and tibias, and 15 × 10^6^ cells were seeded into 100-mm culture dishes (Corning, Tewsburg, MA, USA) at 37°C under 5% CO_2_ conditions. Nonadherent cells were removed after 48 h and attached cells were maintained for 16 days in alpha minimum essential medium (α-MEM; Invitrogen, Grand Island, NY, USA) supplemented with 20% fetal bovine serum (FBS; Equitech-Bio, Kerrville, TX, USA), 2 mM l-glutamine, 55 μM 2-mercaptoethanol, 100 U/ml penicillin, and 100 μg/ml streptomycin (Invitrogen). Colony-forming attached cells were passed once for further experimental use. For colony-forming unit-fibroblastic (CFU-F) assays, 1 × 10^6^ ANCs from bone marrow were seeded into 60-mm culture dishes. After 16 days, the cultures were washed by PBS and stained with 1% toluidine blue solution with 2% paraformaldehyde. Clusters with more than 50 cells were counted as colonies under microscopy.

### Cell proliferation assay

Mouse BMMSCs (10 × 10^3^/well) were seeded on 2-well chamber slides (Nunc, Rochester, NY, USA) and cultured for 2–3 days. The cultures were incubated with BrdU solution (1:100) (Invitrogen) for 20 h, and stained with a BrdU staining kit (Invitrogen) according to the manufacturer's instructions. The samples were then stained with hematoxylin. BrdU-positive and total cell numbers were counted in 10 images per subject. The number of BrdU-positive cells was indicated as a percentage of the total cell number. The BrdU assay was repeated on three independent samples for each experimental group.

### BMMSC surface molecules analysis

WT BMMSCs or *TERT*^−*/*−^ BMMSCs (0.2 × 10^6^) were incubated with 1 μg of PE-conjugated antibodies or isotype-matched control IgGs (Southern Biotech) at 4°C for 30 min. After washing with PBS with 2% FBS and 2% paraformaldehyde fixation, samples were analyzed by FACS^Calibur^ flow cytometry with CellQuest software (BD Bioscience).

### *In vitro* osteogenic differentiation assay

BMMSCs and *TERT*^−*/*−^ BMMSCs were cultured under osteogenic culture conditions in medium containing 2 mM β-glycerophosphate (Sigma-Aldrich), 100 μM l-ascorbic acid 2-phosphate and 10 nM dexamethasone (Sigma-Aldrich). After 4 weeks of induction, the cultures were either stained with alizarin red for mineralized nodule formation or lysed for RNA isolation to identify osteogenic gene expression.

### *In vitro* adipogenic differentiation

For adipogenic induction, 500 nM isobutylmethylxanthine (Sigma-Aldrich), 60 μM indomethacin (Sigma-Aldrich), 500 nM hydrocortisone (Sigma-Aldrich), 10 μg/ml insulin (Sigma-Aldrich), and 100 nM l-ascorbic acid phosphate were added into the growth medium. After 7 days, the cultured cells were stained with Oil Red-O (Sigma-Aldrich), and positive cells were quantified under microscopy and shown as a percentage of the total cells.

### RT-PCR analysis and real-time PCR

After extraction of total RNA (Qiagen, Germantown, MD, USA), cDNA synthesis (Invitrogen), RT-PCR, and TERT real-time PCR were performed according to the manufacturers' instructions. The primers used were *runx2*: forward, 5′-CCGCACGACAACCGCACCAT-3′ and reverse, 5′-CGCTCCGGCCCACAAATCTC-3′; *ocn*: forward, 5′-AAGCAGGAGGGCAATAAGGT-3′ and reverse, 5′-AGCTGCTGTGACATCCATAC-3′; *ppar*γ*2*: forward, 5′-GCTGTTATGGGTGAAACTCTG-3′ and reverse, 5′-ATAAGGTGGAGATGCAGGTTC-3′; *lpl*: forward, 5′-GGGCTCTGCCTGAGTTGTAG-3′ and reverse, 5′-AGAAATTTCGAAGGCCTGGT-3′; *tert*: forward, 5′-GGATTGCCACTGGCTCCG-3′ and reverse, 5′-TGCCTGACCTCCTCTTGTGAC-3′; and *gapdh*: forward, 5′-CACCATGGAGAAGGCCGGGG-3′ and reverse, 5′-GACGGACACATTGGGGGTAG-3′.

### Western blotting analysis

Cells were lysed in M-PER mammalian protein extraction reagent (Thermo, Waltham, MA, USA) with protease and phosphatase inhibitors (Roche, Indianapolis, IN, USA), and proteins were quantified using a protein concentration assay (Bio-Rad Laboratories, Hercules, CA, USA). For TERT experiments, nuclear protein was extracted by NE-PER Nuclear Extraction Kit (Thermo). Twenty micro gram of total or nuclear proteins were separated by SDS-PAGE and transferred to 0.2 μm nitrocellulose membrane. The membranes were blocked with 5% non-fat dry milk and 0.1% Tween-20 for 1 h, followed by incubation overnight with the primary antibodies diluted in blocking solution according to manufacturer's instructions. The membranes were then incubated with primary antibodies overnight, followed by 1 h incubation in HRP-conjugated secondary antibody diluted at 1:10,000 in blocking solution. Immunoreactive proteins were detected using SuperSignal® West Pico Chemiluminescent Substrate (Thermo) and BioMax film (Kodak, Rochester, NY, USA). For TERT experiments, SuperSignal West Femto Chemiluminescent Substrate (Thermo) was used. The intensity of bands was measured by using NIH ImageJ software and normalized to β-Actin.

### T-lymphocytes apoptosis assay

WT BMMSCs or TERT^−/−^ BMMSCs (0.2 × 10^6^) were seeded on a 24-well culture plate (Corning) containing Dulbecco's Modified Eagle's Medium (DMEM; Lonza, Basel, Switzerland) with 10% heat-inactivated FBS, 50 μM 2-mercaptoethanol, 10 mM HEPES, 1 mM sodium pyruvate (Sigma-Aldrich), 1% non-essential amino acid (Cambrex, East Rutherford, NY, USA), 2 mM l-glutamine, 100 U/ml penicillin and 100 mg/ml streptomycin. After incubation for 24 h, T-lymphocytes (1 × 10^6^) from spleen, prestimulated with plate-bound anti-CD3ε (3 μg/ml) and soluble anti-CD28 (2 μg/ml) antibodies, were directly loaded onto BMMSCs and cocultured for 2 days. Apoptotic T cells were detected by staining with CD3 antibody, followed by AnnexinV Apoptosis Detection Kit I (BD Bioscience) and then analyzed by FACS^Calibur^ flow cytometer with CellQuest software. The Transwell system (Corning) was used as a negative control. 0.2 × 10^6^ of BMMSCs were seeded in the lower chambers. Prestimulated T-lymphocytes (1 × 10^6^) were loaded in the upper chambers. After coculture for 2 days, T cells were harvested, stained, and analyzed as described above.

### *In vitro* CD4^+^CD25^+^Foxp3^+^ Treg cell induction

To avoid natural Treg (nTreg) population in this inductive experiment, CD4^+^CD25^−^ T-lymphocytes (1 × 10^6^/well) isolated from splenocytes using a CD4^+^CD25^+^ regulatory T-cell Isolation kit (Miltenyi Biotec, Auburn, CA, USA) were prestimulated with plate-bounded anti-CD3ε antibody (3 μg/ml) and soluble anti-CD28 antibody (2 μg/ml) for 2 days. The activated T-lymphocytes were loaded on a culture of 0.2 × 10^6^ WT BMMSCs or *TERT*^−*/*−^ BMMSCs with recombinant human TGFβ (2 ng/ml) (R'D Systems, Minneapolis, MN, USA) and recombinant mouse IL-2 (2 ng/ml) (R'D Systems). After 3 days, cells in suspension were collected and stained with anti-CD4-PerCP, CD8a-FITC, and CD25-APC antibodies (1 μg each) for 30 min on ice under dark conditions, followed by anti-Foxp3-PE antibody staining using a Foxp3 staining buffer kit (eBioscience) for cell fixation and permeabilization. The cells were analyzed by the FACS^Calibur^ flow cytometer with CellQuest software.

### Allogenic mouse MSCT into Tsk/^+^ mice

Under general anesthesia, WT BMMSCs or *TERT*^−*/*−^ BMMSCs (0.2 × 10^6^ cells/mouse) were infused into Tsk/^+^ mice via the tail vein at 8 weeks of age. For reduced-dose MSCT or Asp-MSCT, 0.02 × 10^6^ cells were used in each Tsk/^+^ mouse. In the disease group, Tsk/^+^ mice received PBS infusion. All mice were sacrificed at 12 weeks of age for further analysis. For measurement of anti-dsDNA antibodies and ANA, peripheral blood serum samples were collected from all experimental mice and analyzed by commercially available enzyme-linked immunosorbent assay (ELISA) kits (Alpha Diagnostics, San Antonio, TX, USA) according to the manufacturer's instructions. For histological assays, skin samples were fixed in 4% paraformaldehyde (Sigma-Aldrich), followed by paraffin embedding. Paraffin sections (6 μm) were stained with hematoxylin and eosin (H'E) and analyzed using NIH ImageJ software.

### RNAi and chemical treatments

BMMSCs (0.5 × 10^6^) were seeded in a 6-well culture plate and treated with *fasl*, *tert*, β*-catenin* siRNAs (Santa Cruz), or the vehicle siRNA control (sc-36869) with lipofectamine reagent (Invitrogen), according to the manufacturers' instructions. After transfection, cells were either used for protein extraction for Western immunoblotting or for *in vitro* co-culture with T- lymphocytes. For chemical reagent treatments, serum-starved BMMSCs were treated with 10 μM β-catenin activator (CHIRON 99021; Chiron Corporation, Emeryville, CA, USA) for 24 h. For Western immunoblotting, BMMSCs were cultured in growth medium with drugs, and protein was extracted using M-PER mammalian protein extraction reagent. For differentiation induction, BMMSCs were cultured under inductive conditions in the presence of drugs (added every 3 days) for 3 weeks, followed by staining and gene expression analysis. 50 μg/mL aspirin was added to MSCs at 50% confluence for 3 days. Aspirin-treated cells were harvested and directly used for further experiments. To analyze the effect of this treatment, cell lysates were harvested at 0, 1, 3, 5, 7, and 14 days post-treatment. In some experiments, after removal of aspirin at day 3, MSCs were cultured for 2 weeks. A TRAP-ELISA assay was performed to detect telomerase activity.

### Measurements of telomerase activity and immunomodulatory factors production

BMMSCs (0.5 × 10^6^/well) were seeded in 6-well culture plates with or without RNAi or chemicals at the indicated concentrations. For the telomerase activity assay, a TeloTAGGG Telomerase PCR ELISA kit (Roche) was used with cell lysates. For chemokine production assays, the supernatant samples from each culture were collected and measured according to manufacturers' instructions, using a Total Nitric Oxide and Nitrate/Nitrite Parameter Assay kit, Prostaglandin E2 Parameter Assay Kit, and Mouse IL-10 Quantikine ELISA Kit (R'D Systems).

### FASL^+^ BMMSCs isolation

Culture expanded BMMSCs were harvested and stained with CD178(FASL)-PE antibody (1 μg antibody for 0.2 × 10^6^ BMMSCs staining) at 4°C for 30 min. After washing by PBS with 2% FBS, samples were sorted by BD FACSAriaII and analyzed by FACS^Calibur^ flow cytometry. After sorting, the FASL^+^ BMMSC population showed greater than 95% expressing FASL, but the FASL^−^ BMMSC population showed only 0.3% expressing FASL.

### Luciferase reporter assay

*fasl*-luciferase promoter reporter constructs were generated by PCR using Pfu polymerase and mouse genomic DNA as a template. Primers containing upstream XhoI and HindIII downstream restriction sites were used to generate *fasl* promoter fragments (1.1 kb construct: forward, 5′-CTCGAGTGTGCTGTGTGATGGTTAAGGCAC-3′ and reverse, 5′-AAGCTTAGCAAGTCCCTACTCCCACG-3′; 2 kb construct: forward, CTCGAGATGGCACTACCAAACTCCAACCCA-3′ and reverse, 5′-AAGCTTAGCAAGTCCCTACTCCCACG-3′). Restriction digested PCR products were subcloned into a pGL3-Basic vector (Promega, Fitchburg, WI, USA). Point mutants were introduced into the reporter by the Pfu/DpnI method. All clones were confirmed by sequencing on both strands. BMMSCs cultured in 6-well plates were co-transfected with 2 μg luciferase reporter and 100 ng Renilla luciferase expression vector to control for transfection efficiency. Forty-eight hours after transfection, cells were lysed in 1× passive lysis buffer, and luciferase activity was measured using the Dual-Glo Luciferase System (Promega) with luminometer (Turner Biosystems, Sunnyvale, CA, USA).

### Chromatin immunoprecipitation assays

Bone marrow mesenchymal stem cells grown in 10 cm cell culture dishes were fixed for 10 min at room temperature by addition of 1% paraformaldehyde to the growth medium. Cells were washed twice in cold PBS supplemented with complete protease inhibitor cocktail and gently scraped from the plate. Cell lysis and chromatin immunoprecipitation were performed using the ChIP Assay Kit (Millipore). For chromatin fragmentation, cells were sonicated using a Branson Sonifier 450 on power setting 4 in 30 s bursts with 1 min cooling on ice for a total sonication time of 4 min. For immunoprecipitation, 1:100 dilutions of TERT or β-catenin polyclonal antibodies were used to capture protein-DNA complexes, and non-specific serum IgG was used as a negative control. All resulting precipitated DNA samples were quantified with real-time PCR and expressed as a percentage of input DNA. The binding site was detected at 1801 bp upstream of the TBE transcription start site, and the region surrounding the binding site was used for amplification (forward primer, 5′-TGTGATTGGTGGACAGTAGGGTGT-3′ and reverse primer, 5′- TGCTCTCCCTGTACCAGATGAGTCTT-3′). For immunoprecipitation-Western blotting, nuclear proteins were extracted, followed by immunoprecipitation with TERT or β-catenin polyclonal antibodies. Captured protein-protein complexes were further analyzed by Western blotting.

### Overexpression of FASL and TERT

Of 293T cells for lentivirus production were seeded in a 10 cm culture dish (Corning) until 80% confluence. Plasmids with the proper proportion (*fasl* and *tert* gene expression vector: psPAX: pCMV-VSV-G (all from Addgene) = 5:3:2) were mixed in opti-MEM (Invitrogen) with Lipofectamin LTX (Invitrogen) according to the protocol of the manufacturer. EGFP expression plasmid (Addgene) was used as a control. The supernatant was collected at 48 h after transfection and filtered through a 0.45 μm filter to remove cell debris. For infection, the supernatant containing lentivirus was added into the target cell culture in the presence of 4 μg/ml polybrene (SIGMA), and the transgene expression was validated by GFP under microscopic observation.

### Statistics

Comparisons between two groups were analyzed by independent two-tailed Student's *t*-tests, and comparisons between more than two groups were analyzed by one-way ANOVA. *P* values <0.05 were considered statistically significant.

**The paper explained**

**Problem**

Autoimmune disease is a major class of human disorders and its treatment remains a clinical challenge. Systemic infusion of bone marrow mesenchymal stem cells (BMMSCs) yields therapeutic benefit for a variety of immune disorders, but the role of the stem cell property in BMMSC-based immunomodulation is poorly understood. Specifically, it is unknown whether unique gene-driven functional commonalities of stem cells play a role in adjusting BMMSC-mediated immune responses. Telomerase plays a crucial role in maintaining BMMSC stemness and ostoegenic differentiation. However, it is unknown whether telomerase activity regulates BMMSC-mediated immune therapies. Elucidating the role of telomerase in BMMSC-based immunomodulation may help to improve BMMSC-mediated immune therapies.

**Results**

Using a genetic TERT null mouse model and TERT knockdown BMMSCs, we showed that *TERT*^−/−^ BMMSCs had reduced immunomodulatory function, suggesting that telomerase activity contributes to BMMSC-mediated immune therapy. Mechanistically, TERT, combined with β-catenin and BRG1, serves as a transcriptional complex binding to the FAS ligand (FASL) promoter to upregulate FASL expression, leading to an elevated immunomodulatory function. Aspirin-pretreated BMMSCs showed elevated telomerase activity and improved immune therapeutic function in treating systemic sclerosis mice.

**Impact**

This study provides the first evidence that TERT regulates the immunomodulatory property of BMMSCs. Elevation of telomerase activity in BMMSCs, as induced by aspirin pretreatment, can improve their immunomodulatory function and reduce their dosage in immune therapy.
