# Supplementary material for: Telomerase governs immunomodulatory properties of mesenchymal stem cells by regulating FAS ligand expression
Source: EMBO Mol Med. 2014 Jan 13;6(3):322–34. doi: 10.1002/emmm.201303000 (PMC3958307; doi:10.1002/emmm.201303000)
Supplement: Supplementary file 1 [file emmm0006-0322-sd1.pdf]

EMBO Molecular Medicine

Telomerase Governs Immunomodulatory Properties of Mesenchymal Stem Cells by Regulating Fas Ligand Expression

Chider Chen, Kentaro Akiyama, Takayoshi Yamaza, Yong-Ouk You, Xingtian Xu, Bei Li, Yimin Zhao, and Songtao Shi

Corresponding author: Songtao Shi, University of Southern California

Review timeline:

Submission date:	04 May 2013
Editorial Decision:	22 July 2013
Additional Editorial correspondance:	14 August 2013
Revision received:	17 October 2013
Editorial Decision:	05 November 2013
Revision received:	10 November 2013
Accepted:	12 November 2013

Transaction Report:

(Note: With the exception of the correction of typographical or spelling errors that could be a source of ambiguity, letters and reports are not edited. The original formatting of letters and referee reports may not be reflected in this compilation.)

Editor: Céline Carret

1st Editorial Decision 22 July 2013

Thank you for the submission of your manuscript to EMBO Molecular Medicine. I am really sorry that it has taken so long to get back to you on your manuscript, which was due to an unfortunate combination of delayed referees and holiday season. Nevertheless, we finally obtained the reports from the two referees who were asked to evaluate the study. Although they find the work to be of potential interest, they also raise a number of concerns that need to be convincingly addressed in the next final version of your article.

As you can see from the enclosed reports, the referees find the topic interesting. While referee 1 is enthusiastic about the findings, together with referee 2 they suggest to perform additional experiments to unequivocally attribute the effect of aspirin on TERT activation to explain the improved immunomodulatory capacities of BMMSCs *in vivo*. In addition, referee 2 raises numerous and important concerns regarding the lack of important controls and details that preclude a proper evaluation of the data.

Given the referees' reports, we would be willing to consider a revised manuscript with the understanding that the reviewers' concerns must be all fully addressed, with additional experiments where appropriate.

In our view the suggested revisions would render the manuscript much more compelling and interesting to a broad readership. We therefore hope that you will be prepared to undertake the recommended experimental revision.

Please note that it is EMBO Molecular Medicine policy to allow a single round of revision in order

to avoid the delayed publication of research findings. Consequently, acceptance or rejection of the manuscript will depend on the completeness of your responses included in the next version of the manuscript.

EMBO Molecular Medicine has a "scooping protection" policy, whereby similar findings that are published by others during review or revision are not a criterion for rejection. Should you decide to submit a revised version, I do ask that you get in touch after three months if you have not completed it, to update us on the status.

Please also contact us as soon as possible if similar work is published elsewhere. If other work is published we may not be able to extend the revision period beyond three months.

***** Reviewer's comments *****

Referee #2 (Comments on Novelty/Model System):

Excellent manuscript with high impact for the therapeutic use of MSC. To determine if the positive effect of aspirin in vivo is truly by activating TERT, an additional experiment could be performed where TERT-/- BMMSC are administered to Tsk/+ mice with or without aspirin. Alternatively, the effect in these mice of Aspirin alone should be evaluated.

Referee #2 (Remarks):

Experiments seem well performed, with only concerns related to flow cytometry data presented in Figure 1C. As presented, it suggests that the population of BMMSC is highly contaminated (over 10%) with other cell types. Is this due the low passage of cells used? An improved FACS analysis is encouraged, to clearly distinguish between "robust expression", "low levels" and "negative" cells. Please indicate number of animals tested (BMMSC donors) and standard deviations. Alternatively, just proof homogeneity of MSC cultures and omit this data (also from discussion) as it seems irrelevant within the context of TERT-mediated immune regulation.

Referee #3 (Comments on Novelty/Model System):

I have many concerns re. the interpretation of the data in this manuscript due to important and relevant controls missing from these studies. I also have many concerns re. the interpretation of some of the data. Please see below.

Referee #3 (Remarks):

In this manuscript the authors claim that TERT forms a complex with B-catenin and BRG1 to regulate Fasl production and thereby induce BMMSC-mediated apoptosis of T cells. While some of these studies show some merit I have numerous concerns re. appropriate controls and also interpretation of the data that need to be addressed.

Major concern:

1) Most controls used in these studies are inadequate. The mouse strains are inappropriately matched (controls for the TERT-/-, Tsk/+ and Faslgld mice should be age-matched (and preferentially sex-matched) littermates from breeding heterozygous lines for each of these strains. The C57BL/6

controls used are not appropriate and may contribute to any differences observed.
2) There are also important controls (or insufficient details of controls) lacking from a number of the figures. What were the vehicle siRNA controls used for the siRNA studies? Single controls (eg of siFASL with no Chir in Fig 3C) are lacking throughout many of the experiments making it impossible to clearly determine the outcomes of these experiments. This is especially of major concern for Figure 4E, in which the siTERT control +BMMSC+T cells without aspirin treatment is missing. When comparing to Figure 1F which shows that siTERT + BMMSCs+ T cells results in reduced T cell apoptosis compared to vehicle, the results of Figure 4E (which shows similar apoptosis compared to vehicle) suggest that aspirin has significant effects on the cells independent of TERT (otherwise the levels should be the same as shown for siTERT in Figure 1F). Aspirin is more classically known for its anti-inflammatory effects, inhibiting COX1 and COX2- how much of the effects observed in response to aspirin are due to these effects rather than TERT, which is less convincing.
3) The authors need to be clearer in details re. some experiments. For example, in the westerns shown they need to clarify which data is from nuclear extracts vs data obtained from whole protein. They do not state which experiments used nuclear vs total protein in any of their studies. This is extremely important for the correct interpretation of the data.
4) Why is TERT protein detectable in TERT-/- cells (Figs 3L and S1A).
5) The amount of aspirin used in these studies is incredibly high. Given that it was added to MSCs at 50% confluence what was the impact of it on the MSC cell proliferation and what influence did this have on the experimental outcomes?
6) Apoptotic cells are AnnexinV+7AAD- whereas those that are AnnexinV+7AAD+ are necrotic. Please revise all figures to reflect the true apoptotic vs necrotic values for each of these studies. Are these studies being performed for long enough to see a true effect as there is not much apoptosis occurring in any of the systems- perhaps a time course would be appropriate to show.
7) I am not convinced that there is a difference in FASL in siB-cat treated cells in Figure 3F. Quantitation of these data are required.
8) How specific are the antibodies the investigators are using- the co-IP study in Figure 3L shows some additional bands that are partially cropped out of the gel- please comment. Furthermore, where was the active B-catenin antibody obtained from?

Minor comments:

1) For the cell proliferation assay, the methods states this was done as a percentage of total cells, yet Figure 1B shows only the number of BrdU+ cells. Please correct for the % of total cells.
2) Given the differences in FACS profiles between the normal and TERT-/- BMMSCs, differentiation capacity etc how can you be confident that all of the effects observed are not merely due to these cells being at different stages of differentiation?
3) Why is there much more B-catenin in the vehicle-treated BMMSCs (Figure 3B) compared to that of BMMSCs in Figure 3A, especially when the amount of protein loaded in Figure 3A seems to be much higher than in Figure 3B. And differences yet again in Figure 3C. How reproducible are these results? Quantitation of these data would be preferred in place of representative gel images.
4) Some of the "control" data in different figures appear identical- ie T cell apoptosis in Figures 1D and F, T cell apoptosis in Figure 1E and G. It is important to clarify whether these were done independently or were indeed the same data.

Additional editorial correspondence 14 August 2013

I have now received a last set of comments from a referee who could not get his/her report on time for the decision to be made.

I copy it here for your information.

I would appreciate if you could take these comments into account while revising your manuscript even though this comes 3 weeks after my decision letter. Would you require additional time to convincingly address all referees' comments please do let us know as soon as you can.

Wishing you all the best for the revisions.

Referee's comments:

The base line of this manuscript is that the authors present an array of non-canonical functions of TERT. It is now well appreciated that besides telomere length maintenance by telomerase, TERT has extra - non-canonical functions. However, it was by no means known that TERT, obviously more than any other protein, regulates that many cellular as well as environmental processes. In itself this is very interesting. However, as presented, this study is missing important controls and TERT needs to be analyzed in much more detail and the different functions of TERT demonstrated much more convincingly.

First, the authors do not relate to the fact that telomerase null mice have no phenotype as long as they still have long telomeres. Only late-generation mice - having experienced short telomeres - show severe phenotypes. Which mice were used ?

Second the authors review that BMMCs loose telomerase activity very rapidly ex vivo - all these basics need to be shown - nevertheless they obviously get all the effects in TERT KO cells as compared to wt cells and relate them to the lack of TERT expression in the KO mice. When was telomerase measured in wt mice? Was TERT expression controlled during the experiments and why do KO cells show a similar faint TERT band as the siRNA treated cells (TERT-/- must lack TERT expression)?

Since the protein level is generally low, RNA analyses may be helpful to control the authors' statements. This is particularly important as telomerase - important for the canonical function of TERT - may not be relevant for the argumentation as telomere length was not investigated, though this may be an important aspect.

Due to the amount of different experiments, the text for the individual parts is very condensed. One therefore would have hoped to get deeper information in the figure legend or specifically in the supplementary data (not word restricted). However, neither the figure legends nor description of the suppl. data help in this respect.

And absolutely cryptic is "We also verified that Asp-BMMSCs from human bone marrow showed increased immunmodulatory properties (S5b)" - the only sentence and information on human cells.

1st Revision - authors' response 17 October 2013

Referee #2 (Comments on Novelty/Model System):
Excellent manuscript with high impact for the therapeutic use of MSC. To determine if the positive effect of aspirin in vivo is truly by activating TERT, an additional experiment could be performed where TERT-/- BMMSC are administered to Tsk/+ mice with or without aspirin. Alternatively, the effect in these mice of Aspirin alone should be evaluated.

Response: We appreciate the reviewer's positive comments and thoughtful suggestion. In this study, BMMSCs were pre-treated with aspirin and subsequently infused into Tsk/+ mice to show increased TERT activity and superior therapeutic effects. According to the reviewer's suggestion, we conducted additional experiments by infusing $TERT^{-/-}$ BMMSCs into Tsk/+ mice with or without aspirin, along with an aspirin-only infusion group, to determine the role of aspirin in BMMSC-mediated immune therapy for Tsk/+ mice. We have added the data in the Results section on page 14: "To confirm that

© EMBO

aspirin-elevated telomerase activity contributes to BMMSC-mediated immune therapy, we infused TERT$^{-/-}$ BMMSCs, with or without aspirin treatment, into Tsk/+ mice and found that they failed to rescue the disease phenotypes, as indicated by no significant changes in terms of the levels of Tregs, Th17, ANA, and dsDNA IgG and IgM antibodies in peripheral blood, tightness of skin, and histological skin hypodermal thickness when compared to the untreated WT BMMSC infusion group (Supplementary Figure S6a-h). In addition, infusion of aspirin alone also failed to rescue the disease phenotypes (Supplementary Figure S6a-h)."

Referee #2 (Remarks):
Experiments seem well performed, with only concerns related to flow cytometry data presented in Figure 1C. As presented, it suggests that the population of BMMSC is highly contaminated (over 10%) with other cell types. Is this due the low passage of cells used? An improved FACS analysis is encouraged, to clearly distinguish between "robust expression", "low levels" and "negative" cells. Please indicate number of animals tested (BMMSC donors) and standard deviations. Alternatively, just proof homogeneity of MSC cultures and omit this data (also from discussion), as it seems irrelevant within the context of TERT-mediated immune regulation.

Response: We thank the reviewer for the constructive comments. According to the reviewer's suggestion, we performed additional experiments and revised Figure 1C-D to improve the FACS analysis. In the previous Figure 1C, we used passage 1 cells. In the new experiments, we used passage 2 cells and performed statistical analysis, from which we present the standard deviation. We have added information regarding the number of animals used in the Figure 1 legend: "Each group of BMMSCs was isolated from 3 independent mice in each independent experiment and the s.d. from the mean values of five independent experiments."

Referee #3 (Comments on Novelty/Model System):
I have many concerns re. the interpretation of the data in this manuscript due to important and relevant controls missing from these studies. I also have many concerns re. the interpretation of some of the data. Please see below.

Referee #3 (Remarks):
In this manuscript the authors claim that TERT forms a complex with B-catenin and BRG1 to regulate Fasl production and thereby induce BMMSC-mediated apoptosis of T cells. While some of these studies show some merit I have numerous concerns re. appropriate controls and also interpretation of the data that need to be addressed.
Major concern:
1) Most controls used in these studies are inadequate. The mouse strains are inappropriately matched (controls for the TERT-/-, Tsk/+ and Faslgld mice should be age-matched (and preferentially sex-matched) littermates from breeding heterozygous lines for each of these strains. The C57BL/6 controls used are not appropriate and may contribute to any differences observed.

Response: We appreciate the reviewer's thoughtful suggestions. We apologize for mislabeling the control groups. In this study, we indeed used age-matched littermates from breeding heterozygous lines for each of these strains as control groups. In the previous version, we labeled controls as WT or C57BL6 due to the fact that the background of all these strains is C57BL6/J. We have revised the manuscript by referring to these animals as "age-matched littermates" instead of "WT or C57BL6". We have added the following sentence in the Materials and Methods section: "Aged-matched female littermates were used in the present study."

2) There are also important controls (or insufficient details of controls) lacking from a number of the figures. What were the vehicle siRNA controls used for the siRNA studies? Single controls (eg of siFASL with no Chir in Fig 3C) are lacking throughout many of the experiments making it impossible to clearly determine the outcomes of these experiments. This is especially of major concern for Figure 4E, in

which the siTERT control +BMMSC+T cells without aspirin treatment is missing. When comparing to Figure 1F which shows that siTERT + BMMSCs+ T cells results in reduced T cell apoptosis compared to vehicle, the results of Figure 4E (which shows similar apoptosis compared to vehicle) suggest that aspirin has significant effects on the cells independent of TERT (otherwise the levels should be the same as shown for siTERT in Figure 1F). Aspirin is more classically known for its anti-inflammatory effects, inhibiting COX1 and COX2- how much of the effects observed in response to aspirin are due to these effects rather than TERT, which is less convincing.

Response: We would like to thank the reviewer for his/her informative comments.

a) The vehicle siRNA control was purchased from Santa Cruz (sc-36869), and consists of a scrambled sequence conjugated to fluorescein. It will not lead to the specific degradation of any cellular message and therefore is suitable as a control to monitor transfection efficiency by fluorescence microscopy. We've added this information in the Material and Methods section.

b) We conducted additional experiments using a siFasL group as a control in Fig 3C and an siTERT group as a control in Fig 4D.

c) We apologize for mislabeling of the Figures. In this study, BMMSCs were pretreated with aspirin and then were used for the experiments. In Figure 4E, the label of aspirin 50 means BMMSC-pretreated with 50 mg/mL aspirin for 3 days, after which we washed out the aspirin, followed by co-culture with activated T cells. The data showed that aspirin-pretreated BMMSCs showed an increased ability to induce T cell apoptosis. We have revised the labels in the Figures as "Asp 50-BMMSC" instead of "Aspirin 50".

d) Since aspirin is a well-known anti-inflammatory drug, we pretreated BMMSCs with aspirin to increase TERT activity and then washed out the aspirin for subsequent *in vivo* cell infusion therapies to avoid aspirin's anti-inflammatory effects. We apologize for the mislabeling on the Figures. In Figure 5, we revised the labels to read "Low Asp-BMMSC" instead of "Low BMMSC+Asp". We have revised the Results section on page 14: "To confirm that aspirin-elevated telomerase activity contributes to BMMSC-mediated immune therapy, we infused $TERT^{-/-}$ BMMSCs, with or without aspirin treatment, into Tsk/+ mice and found that they failed to rescue the disease phenotypes, as indicated by no significant changes in terms of the levels of Tregs, Th17, ANA, and dsDNA IgG and IgM antibodies in peripheral blood, tightness of skin, and histological skin hypodermal thickness when compared to BMMSC infusion group (Supplementary Figure S6a-h). In addition, infusion of aspirin alone also failed to rescue the disease phenotypes (Supplementary Figure S6a-h)."

3) The authors need to be clearer in details re. some experiments. For example, in the westerns shown they need to clarify which data is from nuclear extracts vs data obtained from whole protein. They do not state which experiments used nuclear vs total protein in any of their studies. This is extremely important for the correct interpretation of the data.

Response: We thank reviewer for the suggestions. We used nuclear protein for TERT Western blot analysis and whole cell lysate for other Western blot experiments. We have clarified this fact in M&M section.

4) Why is TERT protein detectable in TERT-/- cells (Figs 3L and S1A).

Response: We thank the reviewer for the comment. The TERT protein level is generally low and we used "SuperSignal West Femto Chemiluminescent Substrate (Thermo Scientific, 34094)." The extremely low levels, if detectable, of TERT protein in $TERT^{-/-}$ cells may due to the background of this highly sensitive substrate. To improve the results of these experiments, we performed additional Western blot experiments on different batches of $TERT^{-/-}$ cells and found no TERT expression. In addition, we performed quantitative PCR to examine the RNA levels of TERT from passage-0 to passage-10 to further confirm our Western blot data, as shown in Supplementary Figure 1A and Figure 3L.

5) The amount of aspirin used in these studies is incredibly high. Given that it was added to MSCs at 50% confluence what was the impact of it on the MSC cell proliferation and what influence did this have on the experimental outcomes?

Response: We appreciate the reviewer for this interesting question. Our data and previous publication (Yamaza et. al., 2008, Plos One) showed that 2.5 and 50 mg/mL aspirin treatment show similar effects in slightly activating TERT in BMMSCs without having a significant effect on BMMSC proliferation. However, these aspirin treatments elevated osteogenic differentiation of BMMSCs (Yamaza et. al., 2008, Plos One). Therefore, the proliferation rate may have no marked influence on our experimental results.

6) Apoptotic cells are AnnexinV+7AAD- whereas those that are AnnexinV+7AAD+ are necrotic. Please revise all figures to reflect the true apoptotic vs necrotic values for each of these studies. Are these studies being performed for long enough to see a true effect as there is not much apoptosis occurring in any of the systems- perhaps a time course would be appropriate to show.

Response: We thank the reviewer for the comments.

a) We used the AnnexinV Apoptosis Detection Kit I (BD Bioscience, Cat# 559763) to detect cell apoptosis. AnnexinV+7AAD- allows investigators to identify early apoptotic cells and AnnexinV+7AAD+ is for late apoptotic and necrotic cells. According to the reviewer's suggestion, we've revised all figures to reflect the early apoptotic vs late apoptotic and necrotic values.

b) As described in the Methods and Materials section, T-lymphocytes from the spleen were pre-stimulated with anti-CD3 and anti-CD28 antibodies for 3 days, and then were directly loaded onto BMMSCs for another 2 days of co-culture. According to the reviewer's suggestion, we performed a time course experiment by co-culturing T cells and BMMSCs for 1, 2, 4, and 6 days to determine the effects of BMMSC-induced T cell apoptosis. The results showed that BMMSCs significantly induced T cell apoptosis (both early stage and late stage) compared to the T cell only group.

7) I am not convinced that there is a difference in FASL in siB-cat treated cells in Figure 3F. Quantitation of these data is required.

Response: We appreciate the reviewer's comment. We redid the Western blot and performed quantitative analysis, as shown in Figure 3F and Supplemental Figure 7.

8) How specific are the antibodies the investigators are using- the co-IP study in Figure 3L shows some additional bands that are partially cropped out of the gel- please comment. Furthermore, where was the active B-catenin antibody obtained from?

Response: We thank reviewer's comment.

1) The additional bands on BRG1 immuno-blotting after IP by b-catenin antibody may due to the non-specific detection of BRG1 antibody, since this non-specific band also can be detected in input samples (as shown in the whole blot picture in Supplemental Figure 7) and the b-catenin antibody (Santa Cruz, sc-7199) is an IP grade antibody.

2) The active b-catenin antibody purchased from Millipore (Cat# 05-665).

Minor comments:
1) For the cell proliferation assay, the methods states this was done as a percentage of total cells, yet Figure 1B shows only the number of BrdU+ cells. Please correct for the % of total cells.

Response: We thank the reviewer for the suggestion. We've corrected the % of total cells.

2) Given the differences in FACS profiles between the normal and TERT-/- BMMSCs, differentiation capacity etc how can you be confident that all of the effects observed are not merely due to these cells being at different stages of differentiation?

Response: We appreciate the reviewer's concern. In this study, we used passages 1 or 2 primary cultured BMMSCs, so we cannot observe any cells undergoing differentiation without specific media induction. Although BMMSCs are a heterogeneous cell population, all of the effects are not due to the cells being at different stages of differentiation.

3) Why is there much more B-catenin in the vehicle-treated BMMSCs (Figure 3B) compared to that of BMMSCs in Figure 3A, especially when the amount of protein loaded in Figure 3A seems to be much higher than in Figure 3B. And differences yet again in Figure 3C. How reproducible are these results? Quantitation of these data would be preferred in place of representative gel images.

Response: We thank the reviewer for the suggestion. Quantitation of all Western data was shown in Supplemental Figure 7. The amount of protein in each independent experiment may be slightly altered, but b-actin expression levels can be used to validate the amount of protein-loading.

4) Some of the "control" data in different figures appear identical- ie T cell apoptosis in Figures 1D and F, T cell apoptosis in Figure 1E and G. It is important to clarify whether these were done independently or were indeed the same data.

Response: We appreciate the reviewer's comment. All data presented in this study were done independently.

Additional referee's comments:

The base line of this manuscript is that the authors present an array of non-canonical functions of TERT. It is now well appreciated that besides telomere length maintenance by telomerase, TERT has extra - non-canonical functions. However, it was by no means known that TERT, obviously more than any other protein, regulates that many cellular as well as environmental processes. In itself this is very interesting. However, as presented, this study is missing important controls and TERT needs to be analyzed in much more detail and the different functions of TERT demonstrated much more convincingly.

First, the authors do not relate to the fact that telomerase null mice have no phenotype as long as they still have long telomeres. Only late-generation mice - having experienced short telomeres - show severe phenotypes. Which mice were used?

Response: We would like to thank the reviewer for the informative comments. A previous report showed that most phenotypes of TERT-/- mice could be observed in fourth generation (G4) mice (Sahin et al., 2011, Nature). In this study, we used a similar strategy to generate $TERT^{+/+}$ (WT) mice by mating heterozygous ($TERT^{+/-}$) pairs and intercrossing with $TERT^{-/-}$ mice to generate G4 $TERT^{-/-}$ mice. BMMSCs were isolated from WT and G4 mice afterward. We have added detailed information regarding the $TERT^{-/-}$ strain maintenance in the Materials and Methods section on page 17: "To maintain the $TERT^{-/-}$ strain and generate $TERT^{+/+}$ (WT) mice, heterozygous ($TERT^{+/-}$) pairs were intercrossed. Also, $TERT^{-/-}$ mice were intercrossed to produce telomerase deficient fourth generation (G4) mice."

Second the authors review that BMMCs loose telomerase activity very rapidly ex vivo - all these basics need to be shown - nevertheless they obviously get all the effects in TERT KO cells as compared to wt cells and relate them to the lack of TERT expression in the KO mice. When was telomerase measured in wt mice? Was TERT expression controlled during the experiments and why do KO cells show a similar faint TERT band as the siRNA treated cells (TERT-/- must lack TERT expression)?

Response: We appreciate the reviewer's comment. Since the TERT protein level is generally low and we used "SuperSignal West Femto Chemiluminescent Substrate (Thermo Scientific, 34094)," the extremely low levels, if detectable, of TERT protein in $TERT^{-/-}$ cells may due to the background of this highly sensitive substrate. To improve the results of these experiments, we performed additional Western blot experiments on different batches of $TERT^{-/-}$ cells and found no TERT expression. In addition, we performed quantitative PCR to examine the RNA level of TERT from passage-0 to passage-10 to further

confirm our Western blot data, as shown in Supplementary Figure 1A. As the data show, TERT expression is maintained at a certain level from P0 to P2 of WT BMMSCs, which were used in this study. However, the expression level of TERT was significantly decreased in passage 5 and undetectable by qPCR in passage 10. On the other hand, TERT expression was undetectable in TERT-/- BMMSCs from p0 to p10.

Since the protein level is generally low, RNA analyses may be helpful to control the authors' statements. This is particularly important as telomerase - important for the canonical function of TERT - may not be relevant for the argumentation, as telomere length was not investigated, though this may be an important aspect.

Response: We thank the reviewer for the comments.

1) According to he reviewer's suggestion, we performed real-time PCR to confirm the TERT expression was controlled during the experiments in this study.

2) As described in M&M section, we used a TeloTAGGG Telomerase PCR ELISA kit (Roche) to measure telomere length, as shown in Figs. 3E, 4A, S1B, S1D, S5A and S5B.

Due to the amount of different experiments, the text for the individual parts is very condensed. One therefore would have hoped to get deeper information in the figure legend or specifically in the supplementary data (not word restricted). However, neither the figure legends nor description of the suppl. data help in this respect. And absolutely cryptic is "We also verified that Asp-BMMSCs from human bone marrow showed increased immunmodulatory properties (S5b)" - the only sentence and information on human cells.

Response: We appreciate the reviewer's constructive comments. We've extensively revised the manuscript according to the reviewers' suggestions. For human BMMSC experiments, we have added the following description in the Results section at page 12-13: "These data suggest that aspirin pretreatment increases telomerase activity and elevates the immunomodulation capacity of mouse BMMSCs. To further extend these findings to clinical application, human BMMSCs were isolated and pretreated with aspirin. We verified that Asp-BMMSCs from human bone marrow showed increased telomerase activity by TRAP-ELISA assay and also show increased TERT expression levels as evaluated by Western blot analysis when compared to the non-pretreated group (Supplementary Figure S5b). To confirm that elevated telomerase activity was related to immunomodulatory properties of Asp-BMMSCs, BMMSC-T cell co-culture experiments were performed to show that Asp-BMMSCs induced increased T cell apoptosis when compared to the BMMSC group (Supplementary Figure S5b)."

Editorial Decision 05 November 2013

Thank you for the submission of your revised manuscript to EMBO Molecular Medicine. We have now received the enclosed reports from the referees that were asked to re-assess it. As you will see the reviewers are now globally supportive and I am pleased to inform you that we will be able to accept your manuscript pending the following final amendments:

1) Provide size bars for the figures 2H and 5H

2) In figure legends, could please amend when appropriate the statistical reference to include in the following format: (test used, n, p-value)

3) We now encourage the publication of source data, particularly for electrophoretic gels and blots, with the aim of making primary data more accessible and transparent to the reader. Would you be willing to provide a PDF file per figure that contains the original, uncropped and unprocessed scans of all or key gels used in figures 3 and 4? The PDF files should be labeled with the appropriate figure/panel number, and should have molecular weight markers; further annotation may be useful but is not essential. The PDF files will be published online with the article as supplementary "Source Data" files. If you have any questions regarding this just contact me.

4) We noticed that two co-authors are listed in the Authors Contribution section to only have commented on the manuscript. We recommend that authorship is restricted to those who have substantially and directly contributed to a study [please check the ICMJE guidelines (http://www.icmje.org/ethical_1author.html)]. In this light, we would like to inquire whether the authorship of SG & YJ is appropriate, or if these people would be better acknowledged in the appropriate section.

5) We would also need a short list of bullet points (up to 5) that summarize the key NEW findings. The bullet points should be designed to be complementary to the abstract and will be used online in our new platform (coming January 2014).

Please submit your revised manuscript within two weeks. I look forward to seeing a revised form of your manuscript as soon as possible.

***** Reviewer's comments *****

Referee #2 (Comments on Novelty/Model System):

In my opinion, the authors addressed both my concerns and the ones from the other reviewers, and have therefore substantially improved the quality of this manuscript. The immunomodulatory properties of MSC are of prime importance. In various countries MSC have been approved by FDA-equivalent insititutions for clinical use in particular for immune modulation purposes. Thus, it is critical to understand the underying molecular mechanisms.

Referee #2 (Remarks):

The authors have addressed all my concerns with appropriate additional experiments. I have no additional comments.

Referee #3:

The revisions provided by the authors have satisfactorily addressed my concerns. The inclusion of human data significantly increased my enthusiasm for this manuscript.

© EMBO
