# Supplementary figures and images for: Telomerase governs immunomodulatory properties of mesenchymal stem cells by regulating FAS ligand expression
Source: EMBO Mol Med. 2014 Jan 13;6(3):322–34. doi: 10.1002/emmm.201303000 (PMC3958307; doi:10.1002/emmm.201303000)

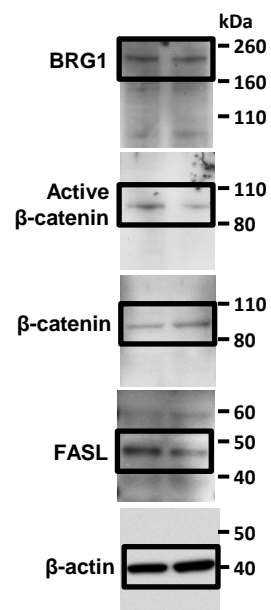

Fig. 3A

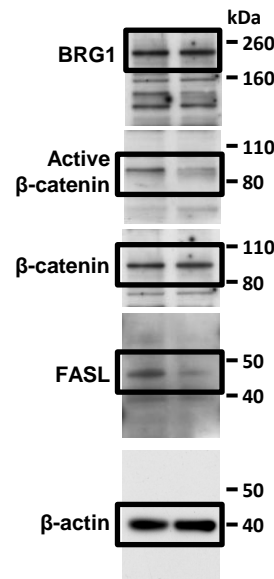

Fig. 3B

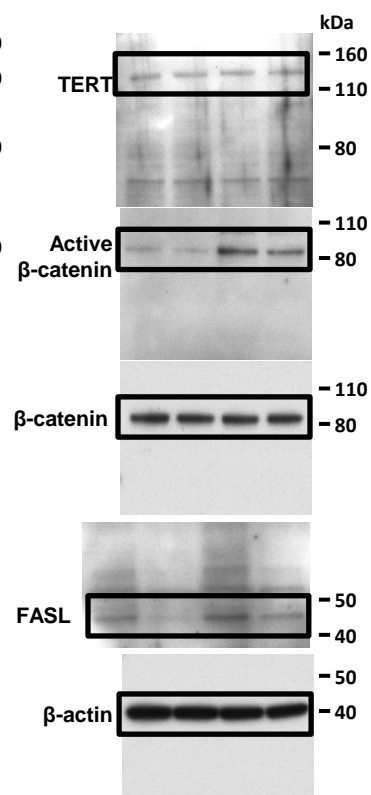

Fig. 3C

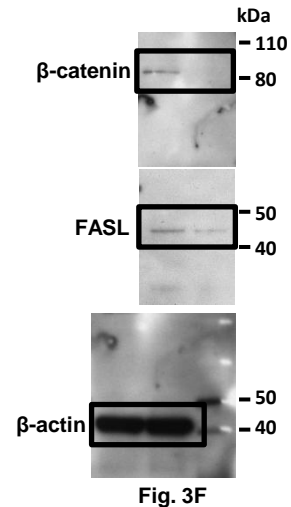

Fig. 3F

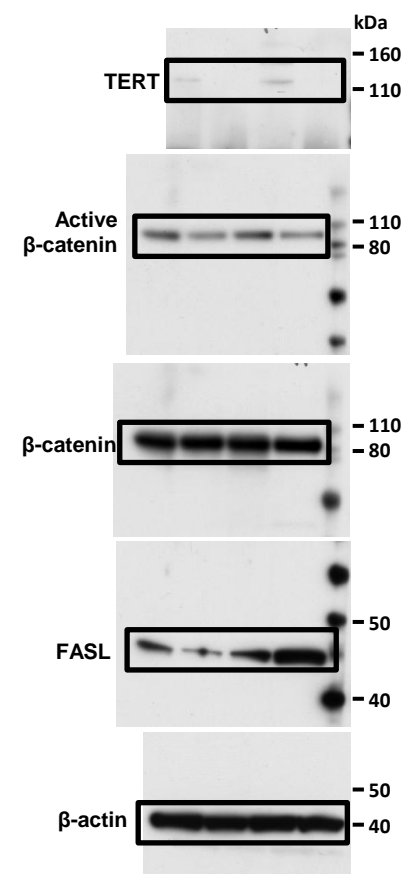

Fig. 3H

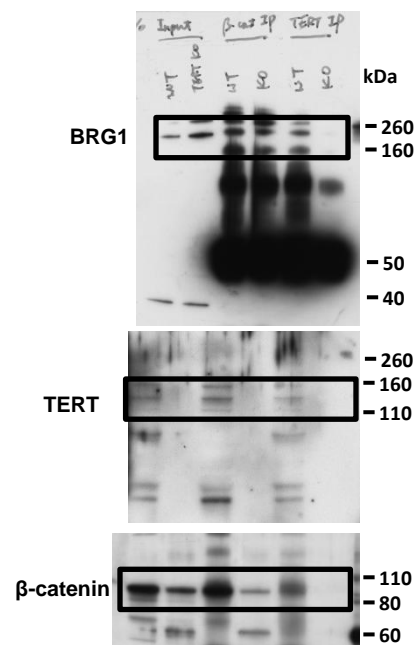

Fig. 3L

Supplement: Supplementary file 2 [file emmm0006-0322-sd2.pdf]

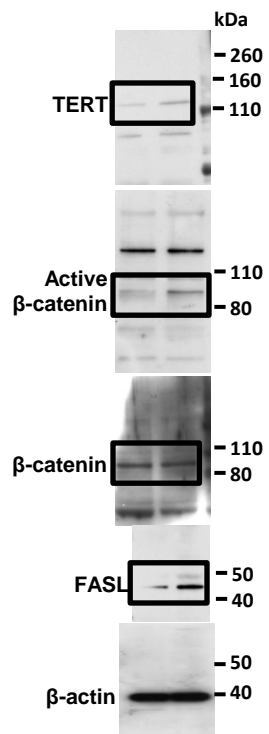

Fig. 4B

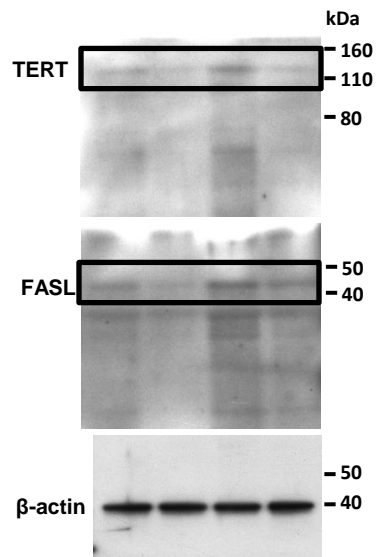

Fig. 4D

Supplement: Supplementary file 3 [file emmm0006-0322-sd3.pdf]
