# Supplementary material for: Telomerase governs immunomodulatory properties of mesenchymal stem cells by regulating FAS ligand expression
Source: EMBO Mol Med. 2014 Jan 13;6(3):322–34. doi: 10.1002/emmm.201303000 (PMC3958307; doi:10.1002/emmm.201303000)
Supplement: Supplementary file 4 [file emmm0006-0322-sd4.pdf]

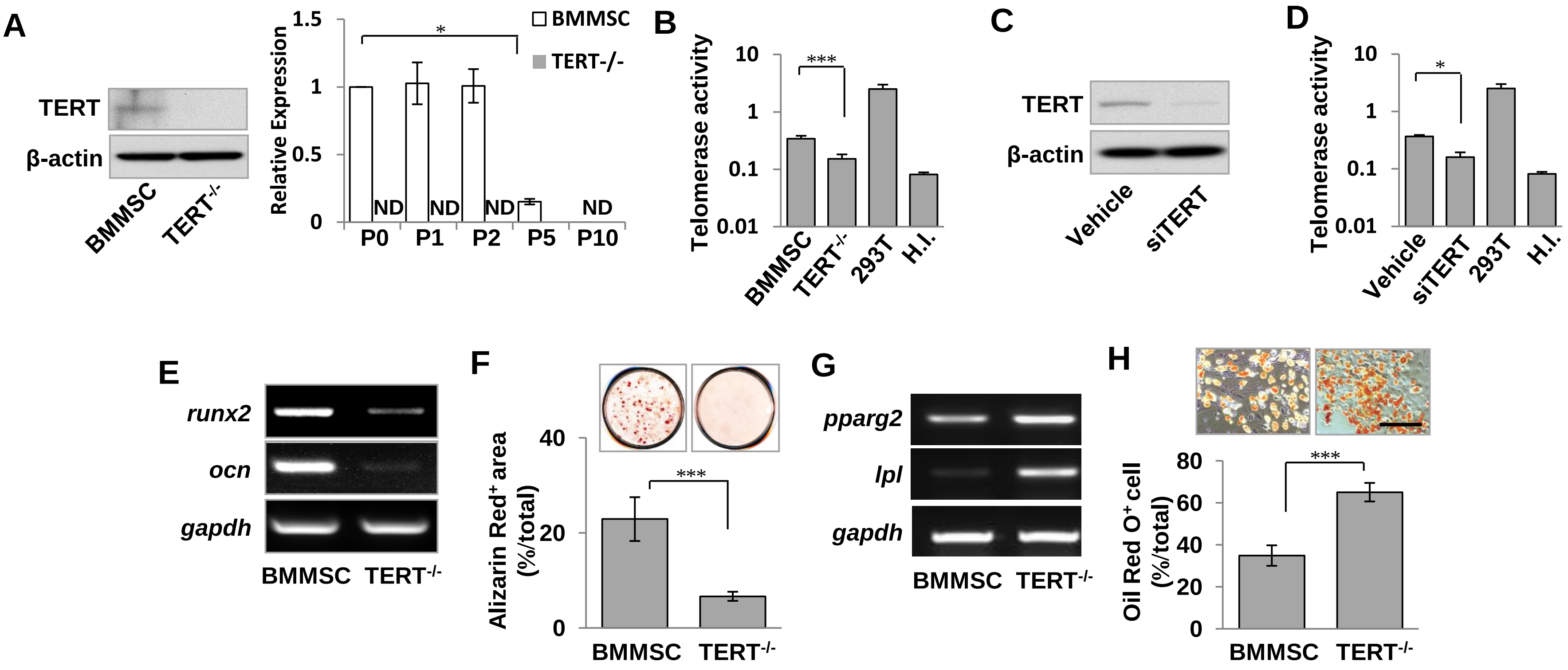

Figure S1 Characterization of BMMSCs from *TERT*$^{-/-}$ mice. (**A**) Western blot showed no TERT expression in *TERT*$^{-/-}$ BMMSCs. Quantitative PCR showed that *TERT* expression was maintained at relatively a high level from P0 to P2 in WT BMMSCs, these cells were used in this study. However, the expression levels of *TERT* were significantly decreased in passage 5 and became undetectable in passage 10. On the other hand, *TERT* expression was undetectable in *TERT*$^{-/-}$ BMMSCs from p0 to p10. (**B**) TRAP-ELISA telomere length analysis showed that *TERT*$^{-/-}$ BMMSCs had no telomerase activity when compared to WT BMMSCs. (**C**) *TERT* siRNA significantly knocked down TERT expression, assessed by Western blot. (**D**) Telomerase activity was significantly decreased in *TERT* siRNA-treated BMMSCs by TRAP-ELISA telomere length analysis. (**E-F**) *TERT*$^{-/-}$ BMMSCs showed downregulation of the osteogenic genes *runx2* and *ocn* (**E**) and bone nodule formation by alizarin red staining (**F**). (**G-H**) *In vitro* adipogenesis evaluation showed that *TERT*$^{-/-}$ BMMSCs had elevated expression of *pparg2* and *lpl* genes (**G**), and Oil red-O staining showed that *TERT*$^{-/-}$ BMMSCs had increased capacity to differentiate into adipocytes (**H**). Vehicle: scrambled siRNA-treated BMMSCs. Error bars present the s.d. from three independent experiments (***p<0.005, *p<0.05).
