# Supplementary material for: Telomerase governs immunomodulatory properties of mesenchymal stem cells by regulating FAS ligand expression
Source: EMBO Mol Med. 2014 Jan 13;6(3):322–34. doi: 10.1002/emmm.201303000 (PMC3958307; doi:10.1002/emmm.201303000)
Supplement: Supplementary file 5 [file emmm0006-0322-sd5.pdf]

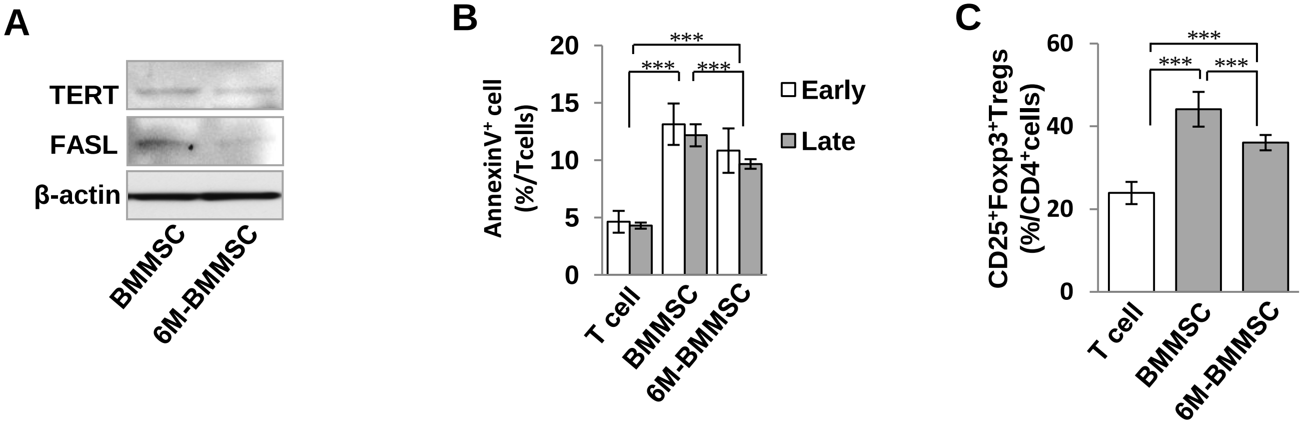

Figure S2 BMMSCs from 6-month-old mice (6M-BMMSCs) have reduced immunomodulatory property. (**A**) TERT and FASL expression levels were significantly decreased in 6M-BMMSCs compared to regular BMMSCs by Western blot analysis. (**B**) BMMSC-induced AnnexinV$^+$7AAD$^-$ and AnnexinV$^+$7AAD$^+$ double positive apoptotic T cells in an *in vitro* co-culture system was dramatically decreased in 6M-BMMSCs. (**C**) *In vitro* Treg induction by 6M-BMMSCs was markedly decreased. Error bars present the s.d. from three independent experiments (***p<0.005).
