# Supplementary material for: Telomerase governs immunomodulatory properties of mesenchymal stem cells by regulating FAS ligand expression
Source: EMBO Mol Med. 2014 Jan 13;6(3):322–34. doi: 10.1002/emmm.201303000 (PMC3958307; doi:10.1002/emmm.201303000)
Supplement: Supplementary file 6 [file emmm0006-0322-sd6.pdf]

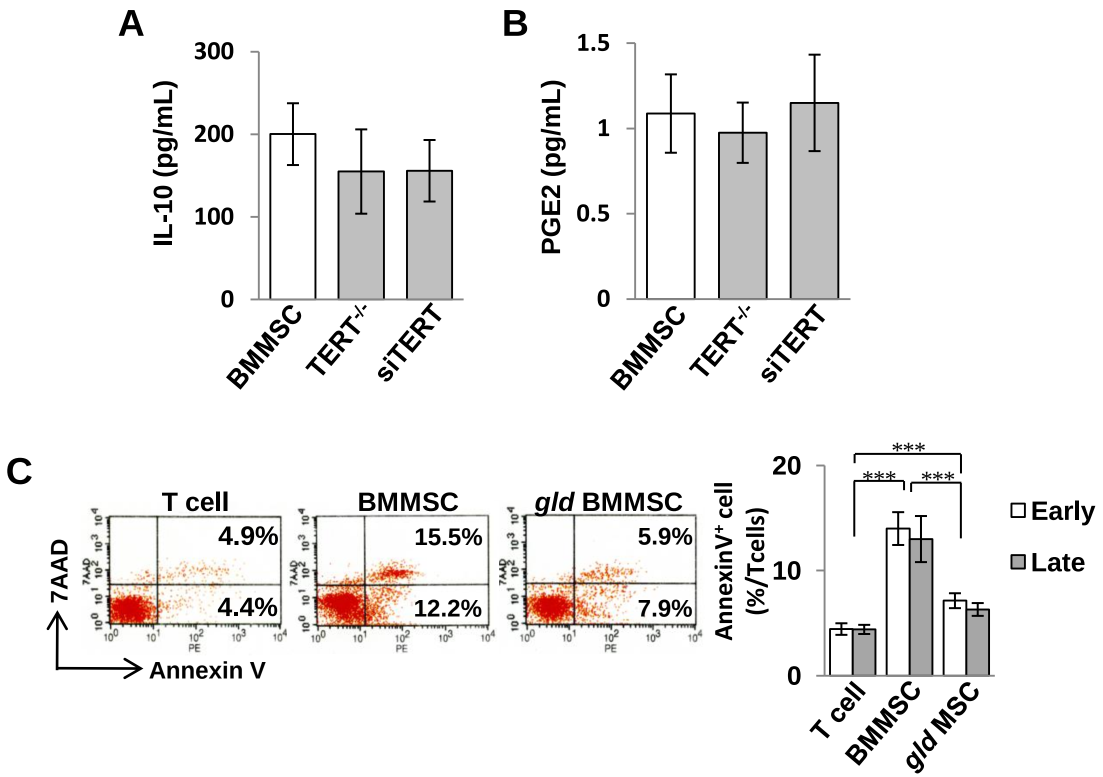

Figure S3 FASL but not immunomodulatory factors mediates immunomodulation of BMMSCs. Knockout of *TERT* or knockdown of *TERT* expression by siRNA in BMMSCs failed to affect expression levels of Interleukin-10 (IL-10) (**A**) and prostaglandin E2 (PGE2) (**B**). (**C**) *In vitro* coculture assay showed that FASL null BMMSCs (*gld*BMMSC) had decreased capacity to induce AnnexinV$^+$7AAD$^-$ and AnnexinV$^+$7AAD$^+$ double positive apoptotic T cells, confirming that FASL is essential for BMMSC-mediated immunomodulation. Error bars present the s.d. from six independent experiments (***p<0.005).
