# Supplementary material for: Telomerase governs immunomodulatory properties of mesenchymal stem cells by regulating FAS ligand expression
Source: EMBO Mol Med. 2014 Jan 13;6(3):322–34. doi: 10.1002/emmm.201303000 (PMC3958307; doi:10.1002/emmm.201303000)
Supplement: Supplementary file 7 [file emmm0006-0322-sd7.pdf]

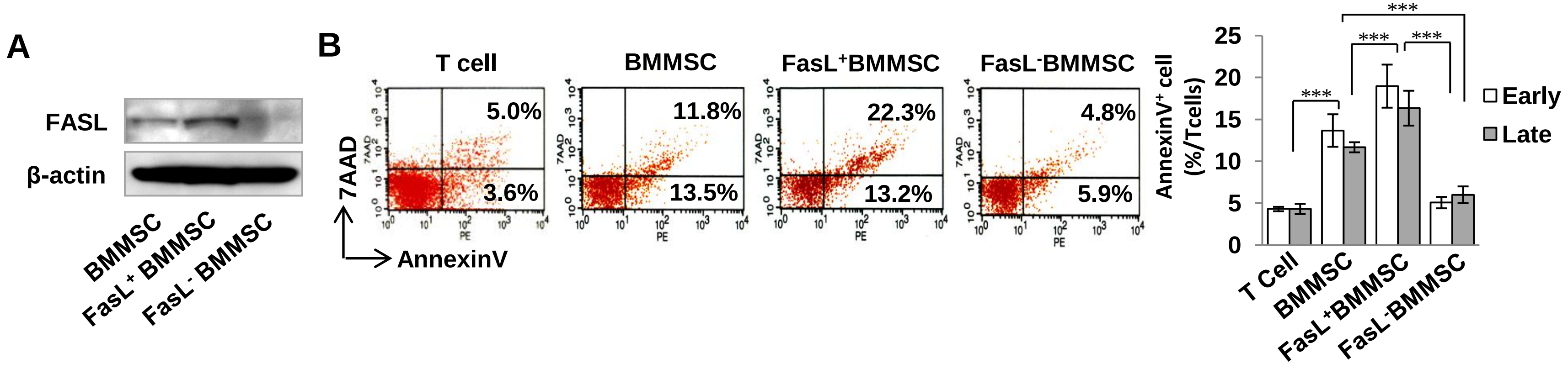

Figure S4 FASL is required for BMMSCs to induce T cell apoptosis *in vitro*. (**A**) Western blot analysis showed that FASL expression level before and after flow cytometric sorting by CD178 (FASL)-PE antibody in BMMSCs. (**B**) $FASL^+$ BMMSCs showed increased capacity to induce $AnnexinV^+7AAD^-$ and $AnnexinV^+7AAD^+$ double positive apoptotic T cells , while $FASL^-$ BMMSCs showed decreased capacity to induce $AnnexinV^+7AAD^-$ and $AnnexinV^+7AAD^+$ double positive apoptotic T cells when compared to non-sorted BMMSCs in an *in vitro* co-culture system. Error bars present the s.d. from six independent experiments (***p<0.005).
