# Supplementary material for: Telomerase governs immunomodulatory properties of mesenchymal stem cells by regulating FAS ligand expression
Source: EMBO Mol Med. 2014 Jan 13;6(3):322–34. doi: 10.1002/emmm.201303000 (PMC3958307; doi:10.1002/emmm.201303000)
Supplement: Supplementary file 8 [file emmm0006-0322-sd8.pdf]

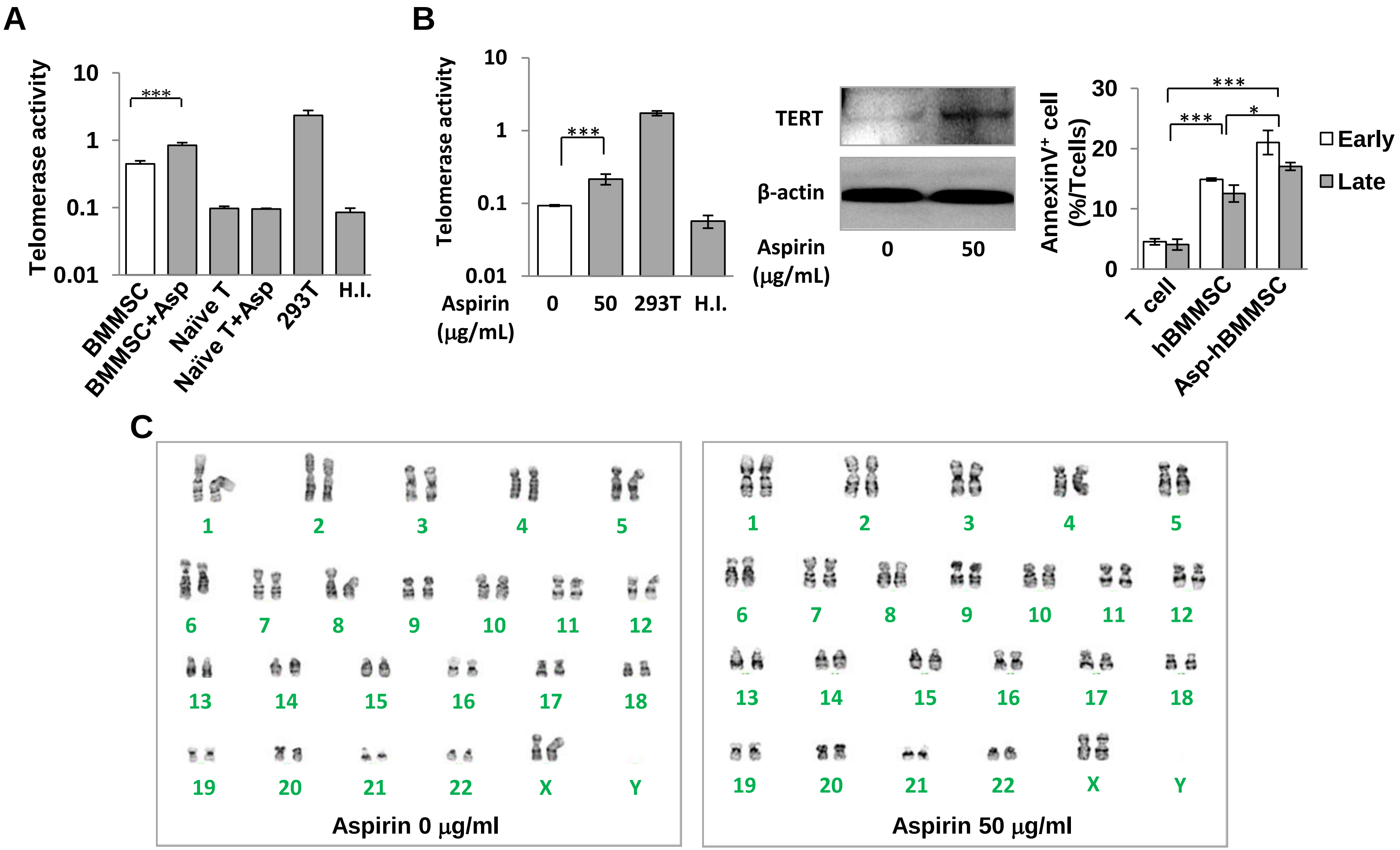

Figure S5 Aspirin pretreatment increased inmmunomodulation of BMMSCs through TERT activation. (**A**) Aspirin treatment showed specific increased telomerase activity in BMMSC, but not naïve T cells. (**B**) Aspirin-treated human BMMSCs showed increased telomerase activity, assessed by TRAP-ELISA telomere length assay. Western blot analysis showed no increased TERT expression in Aspirin-treated human BMMSCs. In an *in vitro* coculture system, aspirin-treated human BMMSCs showed increased capacity in inducing AnnexinV$^+$7AAD$^-$ and AnnexinV$^+$7AAD$^+$ double positive apoptotic T cells. (**C**) there is no karyotype alteration after aspirin treatment compared to untreated group. Error bars present the s.d. from three independent experiments (***p<0.005, *p<0.05).
