# Supplementary material for: Telomerase governs immunomodulatory properties of mesenchymal stem cells by regulating FAS ligand expression
Source: EMBO Mol Med. 2014 Jan 13;6(3):322–34. doi: 10.1002/emmm.201303000 (PMC3958307; doi:10.1002/emmm.201303000)
Supplement: Supplementary file 9 [file emmm0006-0322-sd9.pdf]

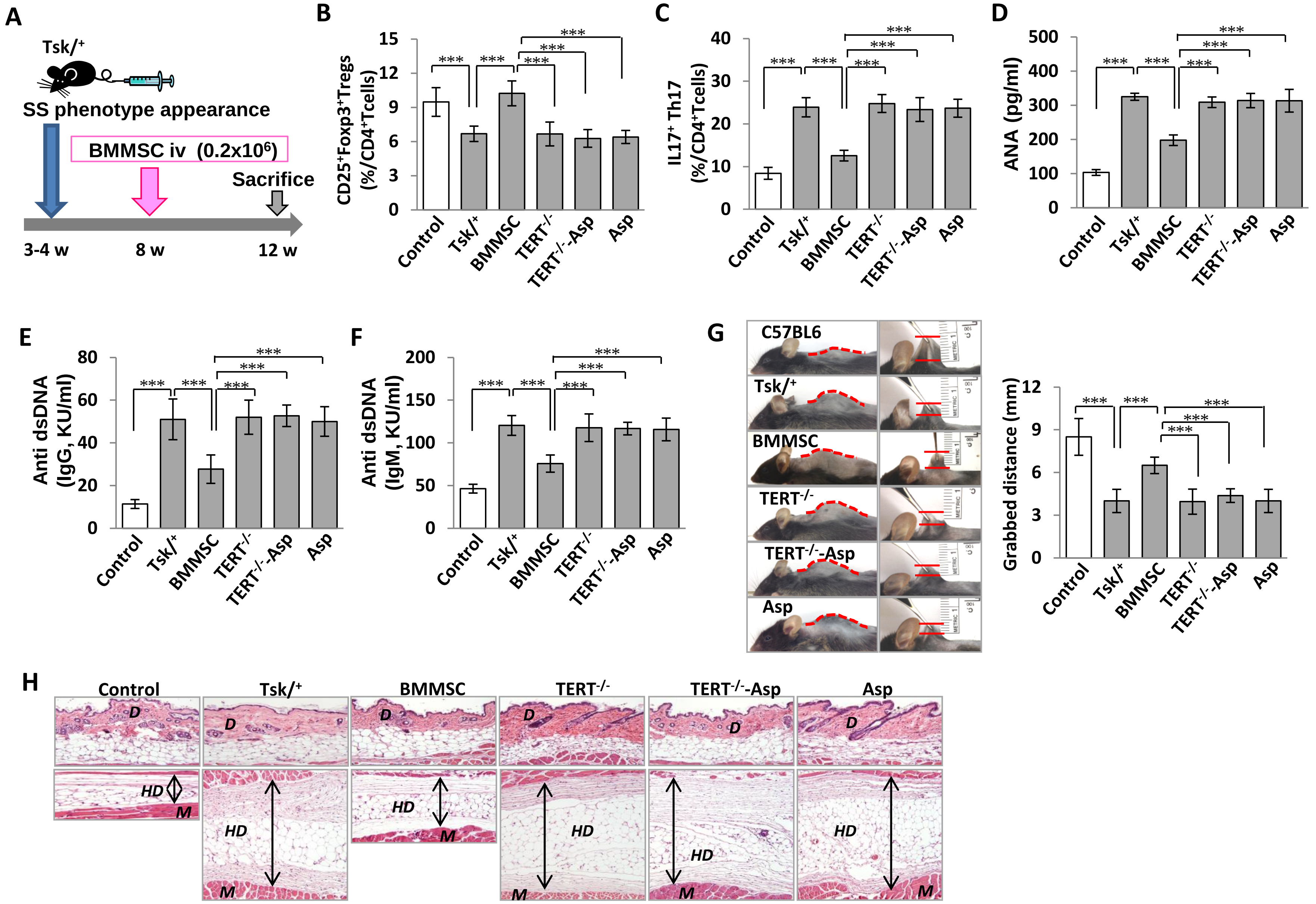
A
Tsk/+
SS phenotype appearance
BMMSC iv (0.2x10^6)
Sacrifice
3-4 w
8 w
12 w
B
CD25+Foxp3+Tregs (%/CD4+Tcells)
C
IL17+ Th17 (%/CD4+Tcells)
D
ANA (pg/ml)
E
Anti dsDNA (IgG, KU/ml)
F
Anti dsDNA (IgM, KU/ml)
G
C57BL6
Tsk/+
BMMSC
TERT-/-
TERT-/--Asp
Asp
Grabbed distance (mm)
H
Control
Tsk/+
BMMSC
TERT-/-
TERT-/--Asp
Asp
D
HD
M
Control
Tsk/+
BMMSC
TERT-/-
TERT-/--Asp
Asp
***

**Figure S6 Aspirin treatment failed to ameliorate systemic sclerosis phenotypes.** (**A**) Schema showed procedure of using *TERT*$^{-/-}$ BMMSCs, aspirin-pretreated *TERT*$^{-/-}$ BMMSCs (*TERT*$^{-/-}$-Asp), or aspirin alone (Asp) to treat systemic sclerosis (SS) tight skin (Tsk/$^{+}$) mice. (**B**) Flow sytometric analysis showed that Treg level was significantly decreased in Tsk/$^{+}$ mice compared to control littermates. After MSCT, Treg level was significantly elevated, whereas *TERT*$^{-/-}$ MSCT failed to upregulate Treg level in Tsk/$^{+}$ mice. *TERT*$^{-/-}$-Asp, along with Asp group, also failed to upregulate Treg levels in Tsk/$^{+}$ mice. (**C**) Flow cytometric analysis showed that CD4$^{+}$IL17$^{+}$ Th17 cells were significantly increased in Tsk/$^{+}$ mice compared to control littermates. MSCT, but not *TERT*$^{-/-}$ MSCT, was able to significantly reduce Th17 level in Tsk/$^{+}$ mice. *TERT*$^{-/-}$-Asp, along with Asp group, also failed to reduce Th17 levels in Tsk/$^{+}$ mice. (**D-F**) ELISA assays showed that Tsk/$^{+}$ mice had elevated levels of anti-double strand DNA antibodies IgG (**D**), IgM (**E**) and antinuclear antibody (ANA, **F**) when compared to control littermates. MSCT reduced the levels of anti-double strand DNA antibodies IgG (**D**), IgM (**E**) and ANA (**F**). In contrast, *TERT*$^{-/-}$ MSCT failed to reduce the levels of anti-double strand DNA antibodies IgG (**D**), IgM (**E**) and ANA (**F**). *TERT*$^{-/-}$-Asp, along with Asp group, also failed to reduce the levels of anti-double strand DNA antibodies IgG (**D**), IgM (**E**) and ANA (**F**). (**G**) Tsk/$^{+}$ mice showed tight skin phenotype. Grabbed skin distance measurement showed that BMMSC, but not *TERT*$^{-/-}$ MSCT, significantly improved tight skin phenotype. *TERT*$^{-/-}$-Asp, along with Asp group, also failed to improved tight skin phenotype. (**H**) Histological examination identified that hyperdermal thickness was significantly increased in Tsk/$^{+}$ mice compared to control littermates. BMMSC, but not *TERT*$^{-/-}$ MSCT improved hyperdermal thickness in Tsk/$^{+}$ mice. *TERT*$^{-/-}$-Asp, along with Asp group, also failed to reduce hyperdermal thickness in Tsk/$^{+}$ mice. D: Dermal, M: Muscle, and HD: Hyperdermal. Error bars represent the s.d. of the mean values (n=6 in each group, ***p<0.005, **p<0.01, *p<0.05).
