# Supplementary material for: Telomerase governs immunomodulatory properties of mesenchymal stem cells by regulating FAS ligand expression
Source: EMBO Mol Med. 2014 Jan 13;6(3):322–34. doi: 10.1002/emmm.201303000 (PMC3958307; doi:10.1002/emmm.201303000)
Supplement: Supplementary file 10 [file emmm0006-0322-sd10.pdf]

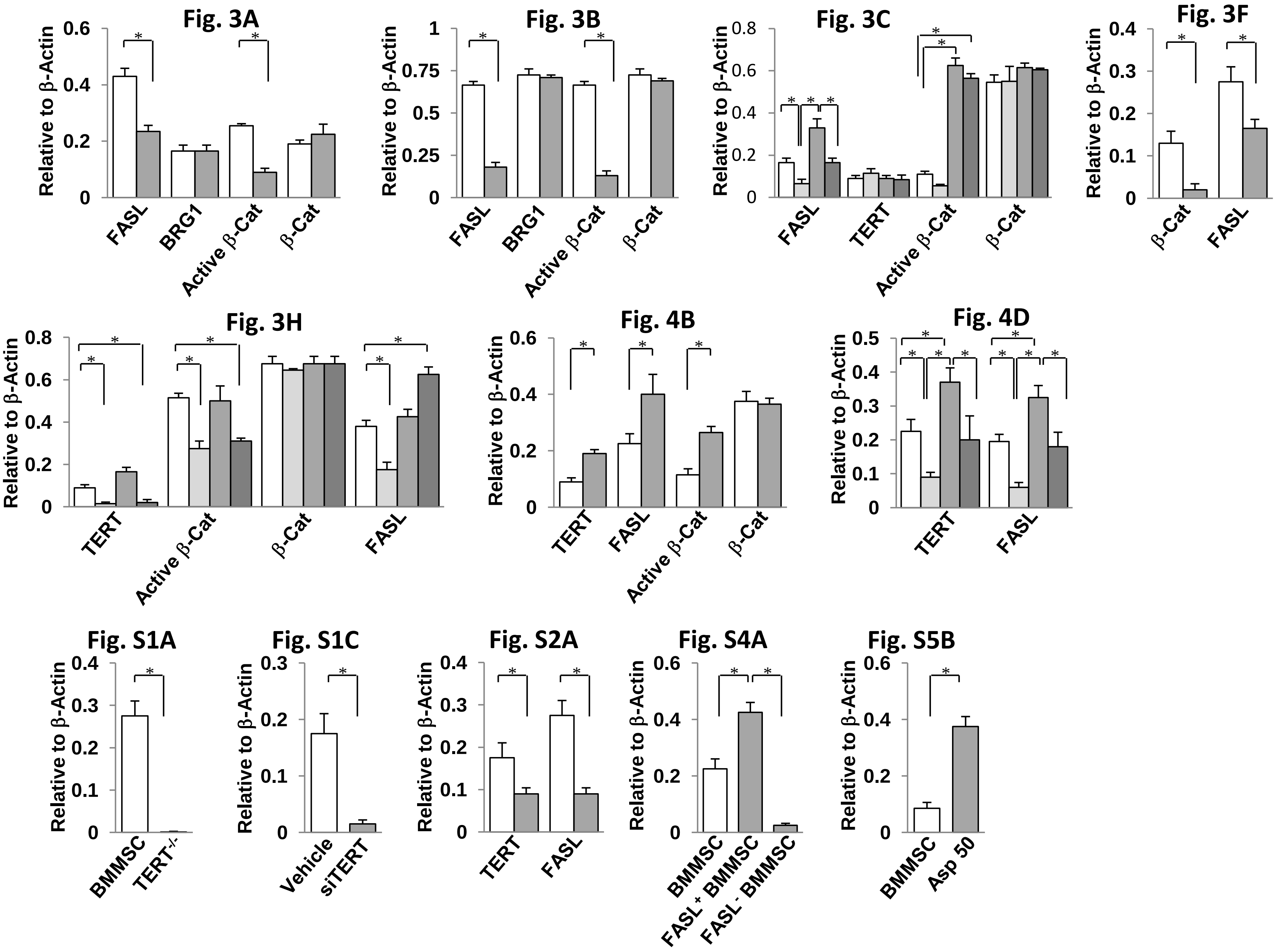

Figure S7 Western blot quantitative analyses. Error bars present the s.d. from three independent experiments (*$p<0.005$).
