# Supplementary material for: Telomerase governs immunomodulatory properties of mesenchymal stem cells by regulating FAS ligand expression
Source: EMBO Mol Med. 2014 Jan 13;6(3):322–34. doi: 10.1002/emmm.201303000 (PMC3958307; doi:10.1002/emmm.201303000)
Supplement: Supplementary file 11 [file emmm0006-0322-sd11.pdf]

Telomerase Governs Immunomodulatory Properties of Mesenchymal Stem Cells by Regulating Fas Ligand Expression

Chider Chen[1,†], Kentaro Akiyama[2,†], Takayoshi Yamaza[3], Yong-Ouk You[1], Xingtian Xu[1], Bei Li[4], Stan Gronthos[5], Yan Jin[4], Yimin Zhao[4*], and Songtao Shi[1*]

Supporting information

Table of Content

Figure S1 Characterization of BMMSCs from *TERT*$^{-/-}$ mice.

Figure S2 BMMSCs from 6-month-old mice have reduced immunomodulatory property.

Figure S3 FASL but not immunomodulatory factors mediates immunomodulation of BMMSCs.

Figure S4 FASL is required for BMMSCs to induce T cell apoptosis *in vitro*.

Figure S5 Aspirin pretreatment increased inmmunomodulation of BMMSCs through TERT activation.

Figure S6 Aspirin treatment failed to ameliorate systemic sclerosis phenotypes.

Figure S7 Western blot quantitative analyses.
